# *N*-Phenyl Cinnamamide Derivatives Protect Hepatocytes against Oxidative Stress by Inducing Cellular Glutathione Synthesis via Nuclear Factor (Erythroid-Derived 2)-Like 2 Activation

**DOI:** 10.3390/molecules26041027

**Published:** 2021-02-15

**Authors:** Sou Hyun Kim, Minwoo Kim, Doyoung Kwon, Jae Sung Pyo, Joo Hyun Kim, Jae-Hwan Kwak, Young-Suk Jung

**Affiliations:** 1 College of Pharmacy, Pusan National University, Busan 46241, Korea; souhyun@pusan.ac.kr (S.H.K.); doyoung.kwon@pusan.ac.kr (D.K.); 2 College of Pharmacy, Kyungsung University, Busan 48434, Korea; kkmmww0902@ks.ac.kr (M.K.); jspyo@ks.ac.kr (J.S.P.); 3 Department of Polymer Engineering, Pukyong National University, Busan 48547, Korea; jkim@pknu.ac.kr

**Keywords:** *N*-phenyl cinnamamide, nuclear factor (erythroid-derived 2)-like 2, antioxidant response element, glutathione

## Abstract

Substituted *N*-phenyl cinnamamide derivatives were designed and synthesized to confirm activation of nuclear factor (erythroid-derived 2)-like 2 (Nrf2) pathway by the electronic effect on beta-position of Michael acceptor according to introducing the R^1^ and R^2^ group. Compounds were screened using the Nrf2/antioxidant response element (ARE)-driven luciferase reporter assay. Compound **1g** showed desirable luciferase activity in HepG2 cells without cell toxicity. mRNA and protein expression of Nrf2/ARE target genes such as NAD(P)H quinone oxidoreductase 1, hemeoxygenase-1, and glutamate-cysteine ligase catalytic subunit (GCLC) were upregulated by compound **1g** in a concentration-dependent manner. Treatment with **1g** resulted in increased endogenous antioxidant glutathione, showing strong correlation with enhanced GCLC expression for synthesis of glutathione. In addition, *tert*-butyl hydroperoxide (*t*-BHP)-generated reactive oxygen species were significantly removed by **1g**, and the results of a cell survival assay in a *t*-BHP-induced oxidative cell injury model showed a cytoprotective effect of **1g** in a concentration dependent manner. In conclusion, the novel compound **1g** can be utilized as an Nrf2/ARE activator in antioxidative therapy.

## 1. Introduction

Oxidative stress, imbalance between reactive oxygen species (ROS) production, and cellular defense capacity lead to chronic diseases including cancer, inflammation, and liver diseases [1,2]. Induction of antioxidant enzymes, including hemeoxygenase-1 (HO-1), NAD(P)H quinone oxidoreductase 1 (NQO1), superoxide dismutase (SOD), catalase, and antioxidant metabolite glutathione (GSH), is considered the primary target for detoxifying ROS [3,4]. They are regulated by the transcription factor nuclear factor (erythroid-derived 2)-like 2 (Nrf2), which is a master regulator of antioxidant response element (ARE)-driven antioxidant gene expression [5]. Cytoprotective effects exerted by natural compounds through induction of antioxidant enzymes by activation of Nrf2 in vitro and in vivo have been reported [6,7,8,9].

Cinnamic acid derivatives are aromatic carboxylic acids (C6–C3) as acrylic acid with a phenyl group at 3-position, found in fruits, vegetables, and beverages such as coffee, tea, and wine. According to substituents on the phenyl group, they are known as cinnamic acid with hydrogen, coumaric acid with a mono-hydroxy group, ferulic acid with a 4-hydroxy-3-methoxy group, and caffeic acid with a 3,4-dihydroxy group. Their various biological activities, including anticancer, antioxidant, antimicrobial, and anti-inflammatory effects, have been reported [1,10,11,12]. Of particular interest, as a natural compound, caffeic acid phenethyl ester (CAPE) has antioxidant effects by activation of the Nrf2-mediated pathway to induce transcription of HO-1. [12]. Ferulic acid ethyl ester (FAEE) has a cytoprotective effect against oxidative damage and amyloid beta(Aβ)-induced oxidative stress in neuronal cells through stimulation of the Nrf2 pathway [13,14]. In addition, it has been reported that compounds such as *N*-cinnamoyl-5-oxo-L-proline methylester (oxoProCA) having cinnamoyl moiety prevents photo-oxidative stress through activation of the Nrf2/ARE pathway against skin cancer cells. They are potent Nrf2-activators and have an α,β-unsaturated carbonyl structure as a pharmacophore, playing a role as an electrophilic Michael acceptor [15]. Several compounds, such as chalcone, dimethyl fumarate, curcumin, and sofalcone, which induce inhibition of Keap1 (Kelch-like ECH-associated protein 1) through a Michael type addition with its cysteine, have been reported to cause activation of the Nrf2/ARE pathway [16].

**Figure 1:**
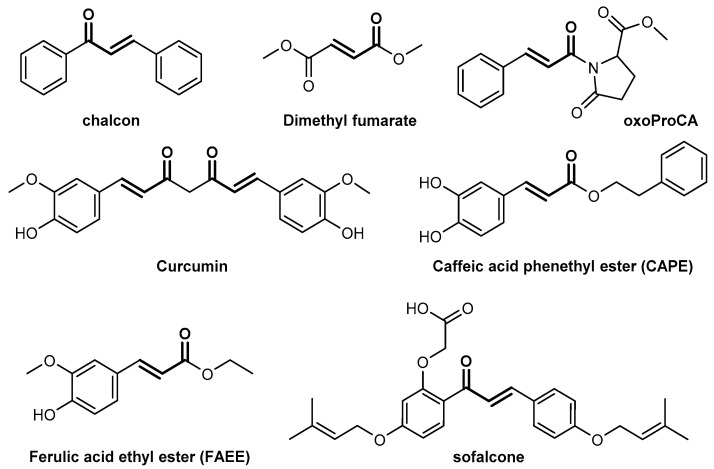
Structure of electrophilic inducers in the nuclear factor (erythroid-derived 2)-like 2 (Nrf2) pathway.

In general, Nrf2 activators, classified as electrophilic activators, have an α,β-unsaturated carbonyl structure as shown in Figure 1. An irreversible covalent bond is formed with the cysteine residue of Keap1 as a major regulator, which modifies its structure followed by activation of Nrf2. However, the electrophilic properties of the beta-position on the carbonyl group have been reported to have adverse effects due to the interaction with the off-target protein at high concentration [16,17,18]. In line with this, the toxicity of chalcone derivatives is altered by modification of the Michael acceptor site [19]. The electron withdrawing group on cinnamamides was also reported to increase Nrf2 transcriptional activity in comparison with *N*-cinnamoyl-L-proline and oxoProCA [15].

**Figure 2:**
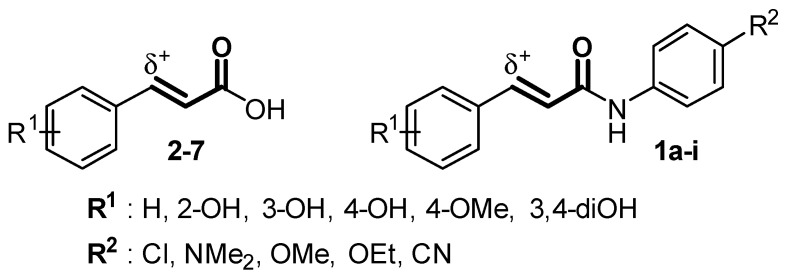
Design of *N*-phenyl cinnamamide derivatives (**1a**–**i**) to confirm electronic effect of their beta-position according to substituents.

In this study, substituted *N*-phenyl cinnamamide derivatives were designed and synthesized by introducing the R^1^ and R^2^ groups for confirmation of Nrf2 activity according to the electronic effect on the beta-position of the Michael acceptor (Figure 2). Biological activity was screened in HepG2 cells using an Nrf2/ARE luciferase reporter assay system. We suggest a novel compound having the highest activity without cytotoxicity as an adverse effect of high concentrations for defense of cells against oxidative stress.

## 2. Results and Discussion

### 2.1. Nrf2/ARE Agonistic Activity of Aromatic Carboxylic Acids (C6–C3)

**Figure 3:**
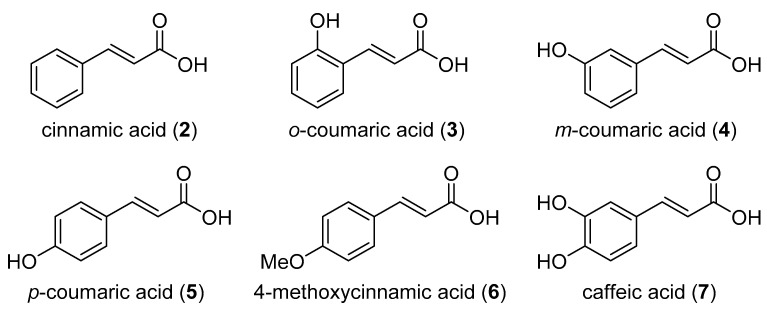
Commercially available cinnamic acid derivatives.

First, six compounds of commercial 3-phenylprop-2-enoic acid derivatives including cinnamic acid (**2**), *o*-coumaric acid (**3**), *m*-coumaric acid (**4**), *p*-coumaric acid (**5**), 4-methoxycinnamic acid (**6**), and caffeic acid (**7**) were prepared (Figure 3). Activity of these compounds in the Nrf2/ARE pathway was determined using a luciferase assay to confirm that biological activity of those containing an α,β-unsaturated carbonyl structure was associated with an electronic effect and lipophilicity of the hydroxy group on the phenyl ring.

According to the result, *m*-coumaric acid (**4**) with a *meta*-hydroxy group, 4-methoxycinnamic acid (**6**) with a *para*-methoxy group, and cinnamic acid (**2**) without an electron-donating group, showed slightly higher activity compared with other compounds (Table 1). On the other hand, compounds (**3**, **5**, and **7**) with a hydroxy group at *ortho*- or *para*-position showed reduced activity of the Nrf2/ARE pathway. Therefore, we confirmed that the electron-donating effect by conjugation of the hydroxy group at the *ortho*- or *para*-position reduces Nrf2/ARE activity through decreasing activity of the Michael acceptor and that physical properties such as lipophilicity also influence the activity in 4-methoxycinnamic acid (**6**), with a cLogP value of 2.16 and compounds (**3** and **5**) with cLogP value of 1.57. In particular, compound **4**, despite having *meta*-hydroxy group, showed the most potent activity among the cinnamic acid derivatives, and the results of a luciferase assay showed a 1.63-fold effect at 5 µM. Although it increases the polarity of molecule, we speculate that the activity of *m*-coumaric acid (**4**) resulted from enhancing the electrophilic effect on the beta-position of the Michael acceptor by electronegative induction of the *meta*-hydroxy group in the comparison with cinnamic acid (**2**). Therefore, substituted *N*-phenyl *m*-coumaric amides and *N*-phenyl cinnamamides were designed and synthesized, and then their agonistic activity of the Nrf2/ARE pathway was evaluated.

### 2.2. Synthesis of Cinnamamide Derivatives

**Scheme 1:**
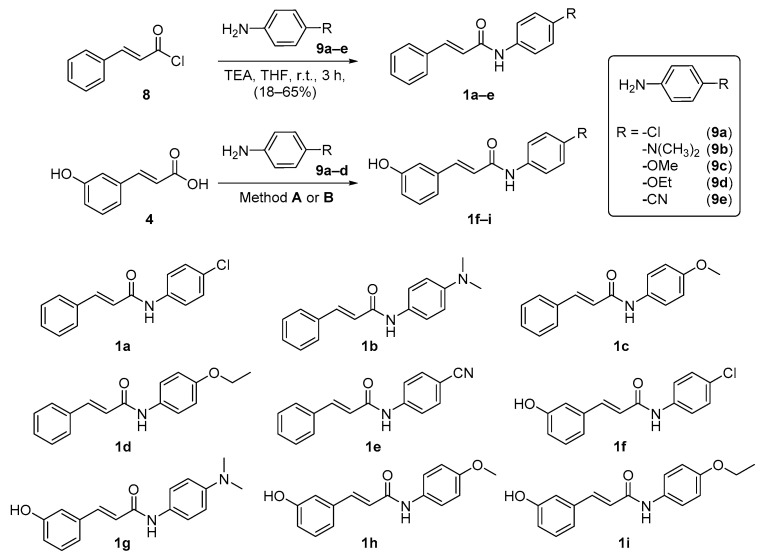
Synthesis of substituted *N*-phenyl cinnamamide derivatives (**1a**–**i**). Reagents and conditions: Method A—SOCl_2_, K_2_CO_3_, THF, then **9a**, TEA, THF, 18 h, 92%; Method B—**9b**–**d**, BOP (benzotriazol-1-yloxytris(dimethylamino)phosphonium hexafluorophosphate), TEA, DCM/DMF, 16–24 h, 28–63%.

To examine the Nrf2/ARE activity of additional compounds, substituted *N*-phenyl cinnamamides were synthesized from cinnamoyl chloride (**8**) and *m*-coumaric acid (**4**) according to the route shown in Scheme 1. For preparation of *N*-phenyl cinnamamide derivatives (**1a**–**e**), commercially available cinnamoyl chloride (**8**) was treated with several substituted aniline and triethylamine in anhydrous tetrahydrofuran. In addition, a similar synthetic method using acid chloride from *m*-coumaric acid (**4**) with thionyl chloride afforded *N*-4-chlorophenyl *m*-coumaric amide (**1f**). *N*-Phenyl *m*-coumaric amide derivatives (**1g**–**i**) were obtained using a BOP (benzotriazol-1-yloxytris(dimethylamino)phosphonium hexafluorophosphate) coupling reagent with several substituted aniline (see Supplementary Materials) [20].

### 2.3. Nrf2/ARE Agonistic Activity of Cinnamamide Derivatives

The synthesized substituted *N*-phenyl cinnamamide derivatives (**1a**–**i**) showed 3.57–15.6 times more potent Nrf2/ARE luciferase activity compared with negative control at 10 µM (Table 1). Compound **1g** showed the highest luciferase activity, as potent as *tert*-butyl hydroquinone (*t*-BHQ), an Nrf2/ARE activator used as positive control. In addition to compound **1g**, compounds possessing *meta*-hydroxy group were tested at various concentrations (1, 3, 5, and 10 µM) for the determination of dose-dependent activity. The results showed that compound **1f** and **1g** significantly increased activity according to dose.

The results confirmed that a substituent R^2^ on *N*-phenyl ring influenced Nrf2/ARE luciferase activity. In the cinnamamide derivatives (**1a**–**1d**), compound **1a**, with a Cl group on the *para*-position of the *N*-phenyl ring, showed the highest activity, a 15.3-fold effect, and compounds **1b** (R^2^: -NMe_2_), **1c** (R^2^: -OMe), **1d** (R^2^: -OEt), and **1e** (R^2^: -CN) had 10.3-, 7.28-, 6.55-, and 3.57-fold effects, respectively. Of particular interest, compounds with a nitrile group as an electron-withdrawing group showed poorer activity than compounds with a dimethylamine, methoxy, or ethoxy group as an electron-donating group at 10 µM. In the *m*-coumaric amide derivatives (**1f**–**i**), compound **1f** (R^2^: -NMe_2_), **1g** (R^2^: -Cl), **1h** (R^2^: -OMe), and **1i** (R^2^: -OEt) had 15.6-, 8.85-, 4.1-, and 3.34-fold effects at 10 µM, respectively. The dimethylamine or chloro group, as a substituent R^2^ on *N*-phenyl ring, induced more potent Nrf2/ARE luciferase activity than the methoxy or ethoxy group, similar to the substituent-dependent activity of the cinnamamide derivatives. Based on the activity of cinnamic acid (**2**) and *m*-coumaric acid (**4**), we suggest that electronegative induction of the *meta*-hydroxy group enhances the electrophilic effect, followed by increasing its activity. On the other hand, the negative role of the hydroxy group at the *meta*-position can be clearly seen by comparing compounds **1f**, **1h,** and **1i** with unsubstituted analogs (**1a**, **1c** and **1d**). Exceptionally, only compound **1g** (15.6-fold), despite including *meta*-hydroxy, exhibited more potent activity of the Nrf2/ARE pathway than compound **1b** (10.3-fold) at 10 µM (Table 2).

### 2.4. Enhancement of Nrf2/ARE-Dependent Gene Expression by Compound **1g** Treatment

**Figure 4:**
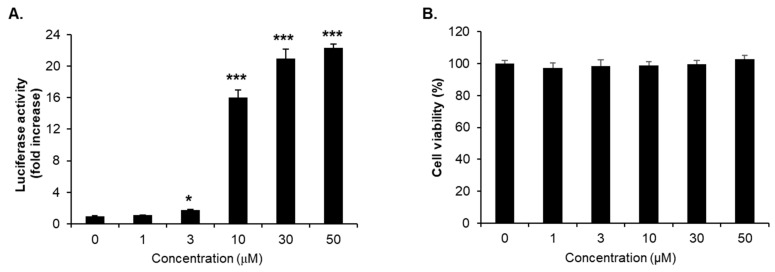
Induction of Nrf2/ARE-directed reporter gene activity by treatment of HepG2-ARE-luciferase cells with compound **1g**. (**A**) Luciferase activity was normalized to total protein and presented as mean fold change ± SD. Each experiment was performed in triplicate and repeated at least three times; representative experimental results are shown. (**B**) Cell viability at the same doses used in a reporter gene assay. * and *** Significantly different from vehicle-treated control cells (0 μM) at *p* < 0.05 and < 0.001, respectively (Student’s *t*-test).

**Figure 5:**
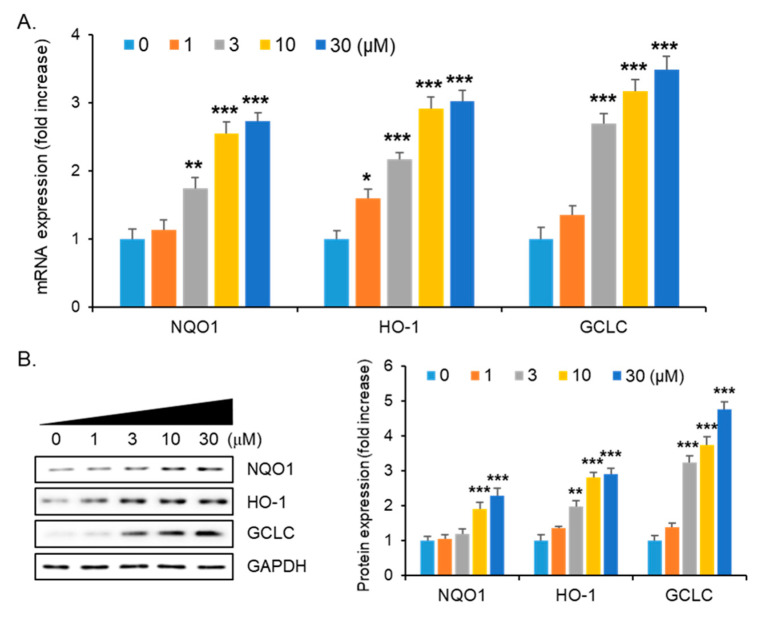
Induction of Nrf2/ARE-dependent genes and protein expression by treatment of HepG2-ARE-luciferase cells with compound **1g**. Expression level of (**A**) mRNA and (**B**) corresponding protein, such as NQO1 (NAD(P)H quinone oxidoreductase 1), hemeoxygenase-1 (HO-1), and glutamate-cysteine ligase catalytic subunit (GCLC). *, **, and *** Significantly different from vehicle-treated control cells (0 μM) at *p* < 0.05, 0.01, and < 0.001, respectively (Student’s *t*-test).

Treatment with compound **1g** resulted in significantly higher Nrf2/ARE luciferase activity compared with other compounds, and more detailed experiments were performed to determine whether it plays a role in cytoprotection against oxidative stress. The concentration-effect relationship of compound **1g** in Nrf2/ARE-driven luciferase activity was examined as shown in Figure 4A. Cell viability was not affected by the same concentration used in the luciferase assay (Figure 4B), indicating that compound **1g** showed no toxicity at these concentrations. In agreement with the effect of compound **1g** on Nrf2/ARE luciferase activity, treatment with compound **1g** resulted in the increased dose-dependent expression of Nrf2-dependent cytoprotective genes (Figure 5A) and their corresponding protein (Figure 5B), such as HO-1 and glutamate-cysteine ligase catalytic subunit (GCLC). These results suggest that compound **1g** could protect cells against oxidative stress.

### 2.5. Protective Effect of Compound **1g** Against Oxidative Cell Death via Increasing GSH Level in HepG2 Cell

**Figure 6:**
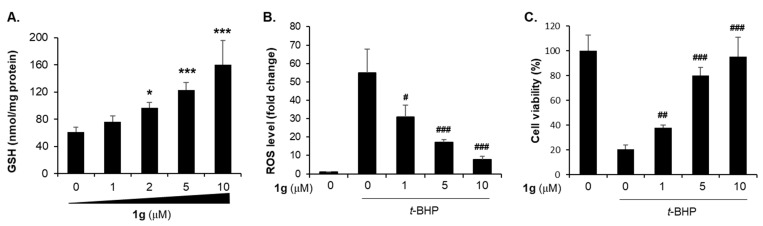
Protective effect of compound **1g** on oxidative stress-mediated cell death. (**A**) Cellular GSH level at 24 h after treatment of HepG2 cells with compound **1g**. (**B**) Reactive oxygen species (ROS) inhibiting effect of compound **1g** in 200 μM of *t*-BHP-treated HepG2 cells. (**C**) Increased cell viability by preincubation of compound **1g** in 200 μM *t*-BHP-treated HepG2 cells. * and *** Significantly different from vehicle-treated control cells (0 μM) at *p* < 0.05 and < 0.001, respectively. ^#^, ^##^ and ^###^ Significantly different from *tert*-butyl hydroperoxide (*t*-BHP)-treated cells at *p* < 0.05, < 0.01, and < 0.001, respectively (Student’s *t*-test).

GSH is an endogenous antioxidant composed of three amino acids, such as glutamate, cysteine, and glycine [21]. GCLC, which is a rate-limiting enzyme in the synthesis of GSH, produces γ-glutamylcysteine, a precursor of GSH [22]. Because treatment with compound **1g** resulted in increased GCLC expression, cellular GSH level was determined by treatment with various concentrations of compound **1g** for 24 h. Treatment with compound **1g** resulted in a significant dose-dependent increase in GSH level (Figure 6A), which showed a correlation with GCLC expression. To examine the protective effect of compound **1g** on *tert*-butyl hydroperoxide (*t*-BHP)-induced oxidative cell death, the cells were pretreated with 1, 5, and 10 μM of compound **1g** for 18 h, followed by incubation with 200 μM *t*-BHP for 24 h. Pretreatment with compound **1g** resulted in a significantly decreased ROS level generated by *t*-BHP (Figure 6B). In addition, treatment at more than 5 μM of compound **1g** completely prevented *t*-BHP-induced oxidative cell death (Figure 6C). These results provide evidence that increased GSH level by treatment with compound **1g** contributes to scavenge *t*-BHP-induced ROS, which protects cells against oxidative stress-mediated cell death.

## 3. Materials and Methods

The chemicals, including acids (**2**–**7**) and *t*-BHQ, were purchased from commercial sources without further purification. THF (tetrahydrofuran) was distilled using sodium and benzopheneone, and DCM (dichloromethane) was distilled using calcium hydride Nuclear magnetic resonance (NMR; ^1^H-NMR and ^13^C-NMR) was measured with a JEOL JNM-EX400 (Tokyo, Japan); chemical shifts are reported in parts per million (ppm, *δ*) and coupling constant is reported in hertz (Hz). Column chromatography isolations were performed using silica gel 60 (230–400 mesh, Merck, Kenilworth, NJ, USA) and medium pressure liquid chromatography (MPLC) system with Biotage Isolera One (Uppsala, Sweden). Analytical thin-layer chromatography (TLC) was performed using a Kieselgel 60 F254 plate (Merck). High resolution mass spectra (HRMS) were expressed in *m*/*z* using the HR-ESI-MS, Agilent 6530 accurate-mass quadrupole time-of-flight (Q-TOF) system (Santa Clara, CA, USA) at the Metabolomics Research Center for Functional Materials, Kyungsung University, Republic of Korea.

### 3.1. Synthesis Procedure for N-Phenyl Cinnamamide Derivatives

#### 3.1.1. General Procedure for 4-Substituted *N*-Phenyl Cinnamamide Derivatives (**1a**–**e**)

Substituted aniline (**9a**–**e**; 1.00 equiv.) and triethylamine (2.00 equiv.) were added to a solution of cinnamolyl chloride (**8**; 1.00 equiv.) in THF. After stirring the mixture at room temperature for 3 h, the solvent was removed in a vacuum, followed by extraction of the residue mixture with EtOAc and 3*N*-HCl. The organic layer was dried using anhydrous magnesium sulfate, and the products (**1a**–**e**) were isolated by column chromatography (EtOAc:hexanes = 1:1~1:2).

##### *N*-(4-chlorophenyl)cinnamamide (**1a**)

Yield: 98 mg (63%); ^1^H-NMR (400 MHz, CDCl_3_) *δ* 7.77 (d, *J* = 15.5 Hz, 1H), 7.56 (m, 4H), 7.36 (m, 6H), 6.53 (d, *J* = 15.5 Hz, 1H); ^13^C-NMR (101 MHz, ACETONE-*D*_6_) *δ* 164.4, 142.0, 139.2, 135.9, 130.7, 129.8, 129.5, 128.7, 128.5, 122.5, 121.5; HRMS (ESI) *m*/*z* [M + H]^+^ calculated for C_15_H_13_ClNO: 258.0680 found: 258.0682.

##### *N*-(4-(dimethylamino)phenyl)cinnamamide (**1b**)

Yield: 29 mg (18%); ^1^H-NMR (400 MHz, CD_3_OD) *δ* 7.58 (m, 3H), 7.47 (m, 2H), 7.38 (m, 3H), 6.76 (m, 3H), 2.89 (s, 6H); ^13^C-NMR (101 MHz, CD_3_OD) *δ* 166.2, 149.5, 142.0, 136.4, 130.9, 130.1, 130.0, 128.9, 122.7, 122.4, 114.4, 41.3; HRMS (ESI) *m*/*z* [M + H]^+^ calculated for C_17_H_19_N_2_O: 267.1492 found: 267.1492.

##### *N*-(4-methoxyphenyl)cinnamamide (**1c**)

Yield: 98 mg (65%); ^1^H-NMR (400 MHz, CDCl_3_) *δ* 7.74 (d, *J* = 15.5 Hz, 1H), 7.53 (m, 5H), 7.36 (m, 3H), 6.87 (d, *J =* 8.9 Hz, 2H), 6.57 (d, *J* = 15.5 Hz, 1H), 3.79 (s, 3H); ^13^C-NMR (101 MHz, CDCl_3_) *δ* 164.1, 156.6, 142.1, 134.8, 131.3, 130.0, 129.0, 128.1, 121.9, 121.0, 114.3, 55.6; HRMS (ESI) *m*/*z* [M + H]^+^ calculated for C_16_H_16_NO_2_: 254.1176 found: 254.1178.

##### *N*-(4-ethoxyphenyl)cinnamamide (**1d**)

Yield: 105 mg (64%); ^1^H-NMR (400 MHz, CD_3_OD) *δ* 7.58 (m, 3H), 7.51 (d, *J* = 9.0 Hz, 2H), 7.37 (m, 3H), 6.85 (d, *J* = 9.0 Hz, 2H), 6.73 (d, *J* = 15.7 Hz, 1H), 3.98 (q, *J* = 7.0 Hz, 2H), 1.34 (t, *J* = 7.0 Hz, 3H); ^13^C-NMR (101 MHz, CDCl_3_) *δ* 164.1, 156.0, 142.0, 134.8, 131.2, 130.0, 129.0, 128.1, 121.9, 121.1, 114.9, 63.8, 15.0; HRMS (ESI) *m*/*z* [M + H]^+^ calculated for C_17_H_18_NO_2_: 268.1332 found: 268.1343.

##### *N*-(4-cyanophenyl)cinnamamide (**1e**)

Yield: 32 mg (21%); ^1^H-NMR (400 MHz, CD_3_OD) *δ* 7.85 (d, *J* = 9.0 Hz, 2H), 7.68 (m, 3H), 7.59 (m, 2H), 7.40 (m, 3H), 6.77 (d, *J* = 15.7 Hz, 1H); ^13^C-NMR (101 MHz, CD_3_OD) *δ* 166.8, 144.6, 143.9, 136.0, 134.3, 131.3, 130.1, 129.1, 121.6, 120.9, 119.9, 107.6; HRMS (ESI) *m*/*z* [M + H]^+^ calculated for C_16_H_13_N_2_O: 249.1022 found: 249.1022.

#### 3.1.2. Procedure for (*E*)-*N*-(4-chlorophenyl)-3-(3-hydroxyphenyl)acrylamide (Method A; **1f**)

Thionyl chloride (2.00 equiv.) was added to a stirred solution of *m*-coumaric acid (1.00 equiv.) in THF [20]. The mixture was stirred under reflux conditions for 12 h, followed by concentration under reduced pressure. The residue was dissolved in THF, 4-chloroaniline (**9a**; 1.00 equiv.), and triethylamine (excess), followed by stirring at room temperature. After 2 h, the product was removed from the solvent under reduced pressure, and compound **1f** was isolated by column chromatography (EtOAc:hexanes = 2:3).

Yield: 154 mg (92%); ^1^H-NMR (400 MHz, CD_3_OD) *δ* 7.65 (d, *J* = 8.9 Hz, 2H), 7.56 (d, *J* = 15.7 Hz, 1H), 7.31 (d, *J* = 8.9 Hz, 2H), 7.21 (t, *J* = 7.8 Hz, 1H), 7.05 (d, *J* = 7.6 Hz, 1H), 6.99 (m, 1H), 6.81 (m, 1H), 6.69 (d, *J* = 15.7 Hz, 1H); ^13^C-NMR (101 MHz, CD_3_OD) *δ* 166.7, 159.1, 143.3, 138.9, 137.4, 131.0, 130.1, 129.8, 122.5, 121.8, 120.5, 118.3, 115.2; HRMS (ESI) *m*/*z* [M + H]^+^ calculated for C_15_H_13_ClNO_2_: 274.0629 found: 274.0628.

#### 3.1.3. General Procedure for 4-Substituted *N*-Phenyl *m*-Coumaric Amide Derivatives (Method B; **1g**–**i**)

A solution of *m*-coumaric acid (1.00 equiv) in DCM was added to TEA (1.00 equiv.), substituted aniline (**9b**–**d**; 1.00 equiv), and BOP ((benzotriazole-1-yloxy)-tris(dimethylamino)phosphonium hexafluorophosphate; 1.00 equiv.) dissolved in DCM at 0 °C [20]. After stirring the mixture at room temperature for 16–24 h, the solvent was removed in a vacuum, followed by extraction using EtOAc and 6*N*-HCl. The organic layer was dried using anhydrous magnesium sulfate, and the products (**1g**–**i**) were isolated by column chromatography (EtOAc:hexanes = 1:2~2:1).

##### (*E*)-*N*-(4-(Dimethylamino)phenyl)-3-(3-hydroxyphenyl)acrylamide (**1g**)

Yield: 58 mg (34%); ^1^H-NMR (400 MHz, CD_3_OD) *δ* 7.51 (d, *J* = 15.6 Hz, 1H), 7.47 (d, *J* = 9.0 Hz, 2H), 7.19 (t, *J* = 7.9 Hz, 1H), 7.03 (d, *J* = 7.8 Hz, 1H), 6.98 (t, *J* = 2.0 Hz, 1H), 6.80 (ddd, *J* = 8.1, 2.5, 0.8 Hz, 1H), 6.76 (d, *J* = 9.1 Hz, 2H), 6.68 (d, *J* = 15.7 Hz, 1H), 2.89 (s, 6H); ^13^C-NMR (101 MHz, CD_3_OD) *δ* 166.3, 159.1, 149.6, 142.2, 137.7, 131.0, 130.1, 122.7, 122.3, 120.4, 118.0, 115.2, 114.4, 41.3; HRMS (ESI) *m*/*z* [M + H]^+^ calculated for C_17_H_19_N_2_O_2_: 283.1441 found: 283.1443.

##### (*E*)-3-(3-Hydroxyphenyl)-*N*-(4-methoxyphenyl)acrylamide (**1h**)

Yield: 88 mg (54%); ^1^H-NMR (400 MHz, CD_3_OD) *δ* 7.53 (d, *J* = 9.2 Hz, 2H), 7.52 (d, *J* = 15.1 Hz, 1H) 7.19 (t, *J* = 7.8 Hz, 1H), 7.03 (d, *J* = 7.7 Hz, 1H), 6.98 (m, 1H), 6.88 (d, *J* = 9.1 Hz, 2H), 6.79 (ddd, *J* = 8.1, 2.4, 0.8 Hz, 1H), 6.68 (d, *J* = 15.6 Hz, 1H), 3.76 (s, 3H); ^13^C-NMR (101 MHz, CD_3_OD) *δ* 166.5, 159.1, 158.0, 142.6, 137.6, 133.0, 131.0, 122.9, 122.1, 120.4, 118.1, 115.2, 115.0, 55.8; HRMS (ESI) *m*/*z* [M + H]^+^ calculated for C_16_H_16_NO_3_: 270.1125 found: 270.1125.

##### (*E*)-*N*-(4-Ethoxyphenyl)-3-(3-hydroxyphenyl)acrylamide (**1i**)

Yield: 64 mg (37%); ^1^H-NMR (400 MHz, CD_3_OD) *δ* 7.50 (d, *J* = 15.8 Hz, 1H), 7.49 (d, *J* = 9.1 Hz, 2H), 7.17 (t, *J* = 7.8 Hz, 1H), 7.01 (d, *J* = 7.7 Hz, 1H), 6.96 (s, 1H), 6.83 (d, *J* = 8.9 Hz, 2H), 6.77 (dd, *J* = 8.0, 2.0 Hz, 1H), 6.66 (d, *J* = 15.6 Hz, 1H), 3.96 (q, *J* = 7.0 Hz, 2H), 1.33 (t, *J* = 7.0 Hz, 3H); ^13^C-NMR (101 MHz, CD_3_OD) *δ* 166.4, 159.1, 157.3, 142.6, 137.6, 132.9, 131.0, 122.9, 122.1, 120.4, 118.1, 115.6, 115.2, 64.7, 15.2; HRMS (ESI) *m*/*z* [M + H]^+^ calculated for C_17_H_18_NO_3_: 284.1281 found: 284.1285.

### 3.2. Evaluation of Biological Activity

#### 3.2.1. Cell Culture

HepG2 human liver cancer cells (Korea Cell Line Bank, Seoul, Korea) were routinely grown in DMEM medium (Hyclone, Logan, UT, USA) supplemented with 10% fetal bo-vine serum (FBS, Hyclone), 2 mM glutamine (Sigma-Aldrich Corp., St. Louis, MO, USA), 100 U/mL penicillin (Hyclone), and 100 μg/mL streptomycin (GenDEPOT, Barker, TX, USA) in a humidified atmosphere at 37 °C with 5% CO_2_.

#### 3.2.2. Nrf2/ARE Luciferase Assay

HepG2 cells were seeded in a 6-well culture plate in antibiotic-free medium for 24 h, followed by transfection with an ARE reporter vector that encodes for firefly luciferase in the presence of Lipofectamine 3000 (Invitrogen, San Diego, CA, USA) according to the manufacturer’s instruction. The cells were then treated with different concentrations of compound **1g** for 6 h. Luciferase Assay Reagent II (Promega, Madison, WI, USA) was added to determine the activity of firefly luciferase.

#### 3.2.3. Cell Viability Assay

The 3-(4,5-dimethylthiazol-2-yl)-2,5-diphenyltetrazolium bromide (MTT) assay, which is a test of normal metabolic status of cells based on the estimated mitochondrial activities, was used for determination of cell viability. HepG2 cells were seeded in a 96-well culture plate at a density of 1.0 × 10^4^ cells/well and incubated overnight. Following pretreatment with different concentrations of compound **1g** for 18 h, cells were treated with *t*-BHP for 24 h and incubated with 0.5 mg/mL MTT reagent at 37 °C for 1 h. Formazan granules generated by live cells were dissolved in dimethyl sulfoxide (DMSO), and the absorbance was measured at 540 nm using the MULTISKAN GO reader (Thermo Scientific, Sunnyvale, CA, USA). Results were expressed as a percentage of viable compound **1g**-treated cells normalized to those of untreated cells.

#### 3.2.4. Real-Time Reverse Transcription-Polymerase Chain Reaction (RT-PCR)

The total RNA was harvested from cells using the Direct-zol RNA kit (Zymo Research, Orange, CA, USA). The cDNA was synthesized by the iScript cDNA Synthesis system (Bio-Rad, Hercules, CA, USA) according to the manufacturer’s protocols. Amplification of transcripts was performed by using the SensiFAST SYBR qPCR mix (Bioline, London, UK). The optimal number of PCR cycles was determined for each primer set to ensure a linear range of amplification. The relative values of gene expression were normalized to 18 s. The primer sequences are provided in Table 3.

#### 3.2.5. Western Blot Analysis

Cells were harvested after treatment of compound **1g**. Total cells were lysed with ice-cold ProEXTM CETi protein extract solution (Translab, Daejeon, Korea), and the concentration of the protein was measured using a BCA reagent (Thermo Scientific). Protein samples were denatured by boiling at 100 °C for 5 min in 4X laemmli sample buffer (Bio-Rad, Hercules, CA, USA) and were separated by sodium dodecyl sulfate (SDS)-polyacrylamide electrophoresis gel. Then it was transferred onto a nitrocellulose (NC) membrane (Bio-Rad, Hercules, CA, USA). The membranes were blocked with TBS-T buffer (Tris-HCl (100 mM, pH 7.5), NaCl (150 mM), and 0.2% Tween-20) containing 5% non-fat dry milk for 1 h at room temperature. Then, the membranes were washed with TBS-T buffer and incubated overnight at 4 °C with the primary antibodies. After washing with TBS-T, the membrane was incubated for 1 h with the appropriate horseradish peroxidase-conjugated secondary antibodies. The blot was detected using EZ-Western Lumi Pico (DOGEN, Seoul, Korea).

#### 3.2.6. GSH Level in HepG2 Cell Line

HepG2 cells were seeded in a 60 mm culture plate for 24 h and then treated with compound **1g** for 18 h. Cells were lysed using a 4-fold volume of ice-cold 1M perchloric acid. After the protein was removed by centrifugation at 10,000× *g* for 10 min, the supernatant was assayed for the total GSH concentration using HPLC [23]. Glutathione was analyzed with *ortho*-phthalaldehyde/2-mercaptoethanol and quantified using an HPLC system equipped with a fluorescence detector (excitation 338 nm and emission 425 nm; FLD-3100, Thermo Scientific) after separation using a Hector T-C18 column (3 µm × 4.6 mm × 100 mm; Chiral Technology Korea, Daejeon, Korea).

#### 3.2.7. Evaluation of ROS Level

To assess cellular ROS levels, HepG2 cells were seeded at 1.0 × 10^4^ cells per well in a 96-well plate. The cells were pre-incubated with compound **1g** for 18 h and then incubated with *t*-BHP for another 4 h to induce the ROS production. ROS-Glo H_2_O_2_ assay system kit (Promega) was used, and it was done following the manufacturer’s instructions. The intensity of the fluorescence in the cells was measured using a Glomax Explorer System (Promega). ROS production was calculated relative to the vehicle-treated control.

#### 3.2.8. Statistical Analysis

All results expressed as the mean ± standard deviation (SD) were analyzed by a two-tailed Student’s *t*-test. The acceptable level of significance was established at *p* < 0.05.

## 4. Conclusions

In summary, nine substituted *N*-phenyl cinnamamide derivatives (**1a**–**i**) were designed and synthesized according to biological evaluation with six cinnamic acid derivatives (**2**–**7**). Especially, substituent R^2^ at *para*-position on *N*-phenyl ring was introduced to confirm activation of Nrf2/ARE pathway by electronic effect on the beta-position of a Michael acceptor. Compounds with a chloro group or dimethylamine group as the R^2^ group showed more potent activity than other compounds. Among them, compound **1g** was screened out to show significant concentration-dependent protection against *t*-BHP-induced HepG2 cell damage. Results of a mechanism study showed that compound **1g** activates the Nrf2 pathway, resulting in increased expression of target genes such as NQO1, HO-1, and GCLC. In addition, an increase in GSH level was observed, reflecting the result of GCLC expression, which is a rate-limiting enzyme in the synthesis of GSH. Considering that GSH is a non-protein thiol for removal of reactive substances, use of compound **1g** will lead to improved endogenous antioxidant capacity, thus preventing cellular damage against oxidative stress.

## Figures and Tables

**Table 1:** Antioxidant response element (ARE) agonistic activity of compounds (**2**–**7**) on a HepG2 cell line.

Entry	Concentration (µM)	Relative Luciferase Activity (Fold)
Con	0	1.00 ± 0.056
Cinnamic acid (**2**)	10	1.22 ± 0.036
*o*-Coumaric acid (**3**)	10	1.16 ± 0.061
*m*-Coumaric acid (**4**)	10	1.45 ± 0.033
*p*-Coumaric acid (**5**)	10	1.12 ± 0.031
4-Methoxycinnamic acid (**6**)	10	1.22 ± 0.058
Caffeic acid (**7**)	10	1.01 ± 0.047
*m*-Coumaric acid (**4**)	1	1.21 ± 0.001
	3	1.32 ± 0.150
	5	1.63 ± 0.207
	10	1.45 ± 0.033

**Table 2:** Relative luciferase activity of *N*-phenyl cinnamamide derivatives (**1a**–**i**) and *tert*-butyl hydroquinone (*t*-BHQ) in a HepG2 cell line.

Entry	Concentration (µM)	Relative Luciferase Activity (Fold)
Con.	0	1.00 ± 0.056
*t*-BHQ	5	5.74 ± 0.439
	10	15.6 ± 0.334
**1a**	10	15.3 ± 0.903
**1b**	10	10.3 ± 0.665
**1c**	10	7.28 ± 0.437
**1d**	10	6.55 ± 0.356
**1e**	10	3.57 ± 0.543
**1f**	1	0.95 ± 0.038
	3	1.18 ± 0.029
	5	3.36 ± 0.653
	10	8.85 ± 1.081
**1g**	1	0.84 ± 0.024
	3	1.50 ± 0.018
	5	5.00 ± 0.488
	10	15.6 ± 1.783
**1h**	1	0.81 ± 0.008
	3	0.90 ± 0.020
	5	1.51 ± 0.154
	10	4.10 ± 0.306
**1i**	1	0.90 ± 0.030
	3	0.91 ± 0.003
	5	1.78 ± 0.217
	10	3.34 ± 0.303

**Table 3:** List of human primer used for real-time reverse transcription-polymerase chain reaction (RT-PCR).

Genes		Primer Sequences
*NQO1*	F: AGGCTGGTTTGAGCGAGT	R: ATTGAATTCGGGCGTCTGCTG
*HO-1*	F: CGGGCCAGCAACAAAGTG	R: AGTGTAAGGACCCATCGGAGAA
*GCLC*	F: TGGCAATGCAGTGGTGGAT	R: AACACACCTTCCTTCCCATTGA
*18s*	F: CAGCCACCCGAGATTGAGCA	R: TAGTAGCGACGGGCGGTGTG

## Data Availability

The data that support the findings of this study are available from the corresponding author upon reasonable request.

## Supplementary Materials

The following are available online. Copies of ^1^H- and ^13^C-NMR spectra of compound **1a**–**i**.

## Sample Availability

Samples of the compounds are available from the corresponding author.
