# Supplementary material for: N-Phenyl Cinnamamide Derivatives Protect Hepatocytes against Oxidative Stress by Inducing Cellular Glutathione Synthesis via Nuclear Factor (Erythroid-Derived 2)-Like 2 Activation
Source: Molecules. 2021 Feb 15;26(4):1027. doi: 10.3390/molecules26041027 (PMC7919495; doi:10.3390/molecules26041027)
Supplement: Supplementary file 1 [file molecules-26-01027-s001.pdf]

Supplementary Material

N-Phenyl cinnamamide derivatives protect hepatocytes against oxidativestress by inducing cellular glutathione synthesis via nuclear factor(erythroid-derived 2)-like 2 activation

Sou Hyun Kim [1,+], Minwoo Kim [2,+], Doyoung Kwon [1,+], Jae Sung Pyo [2], Joo Hyun Kim [3], Jae-Hwan Kwak [2,*], Young-Suk Jung [1,*]

[1]College of Pharmacy, Pusan National University, Busan, 46241, Republic of Korea
[2]College of Pharmacy, Kyungsung University, Busan, 48434, Republic of Korea
[3]Department of Polymer Engineering, Pukyong National University, Busan, 48547, Republic of Korea

*Correspondence: jhkwak@ks.ac.kr (J.-H.K.); youngjung@pusan.ac.kr (Y.-S.J.)
+ These authors contributed equally to this work.

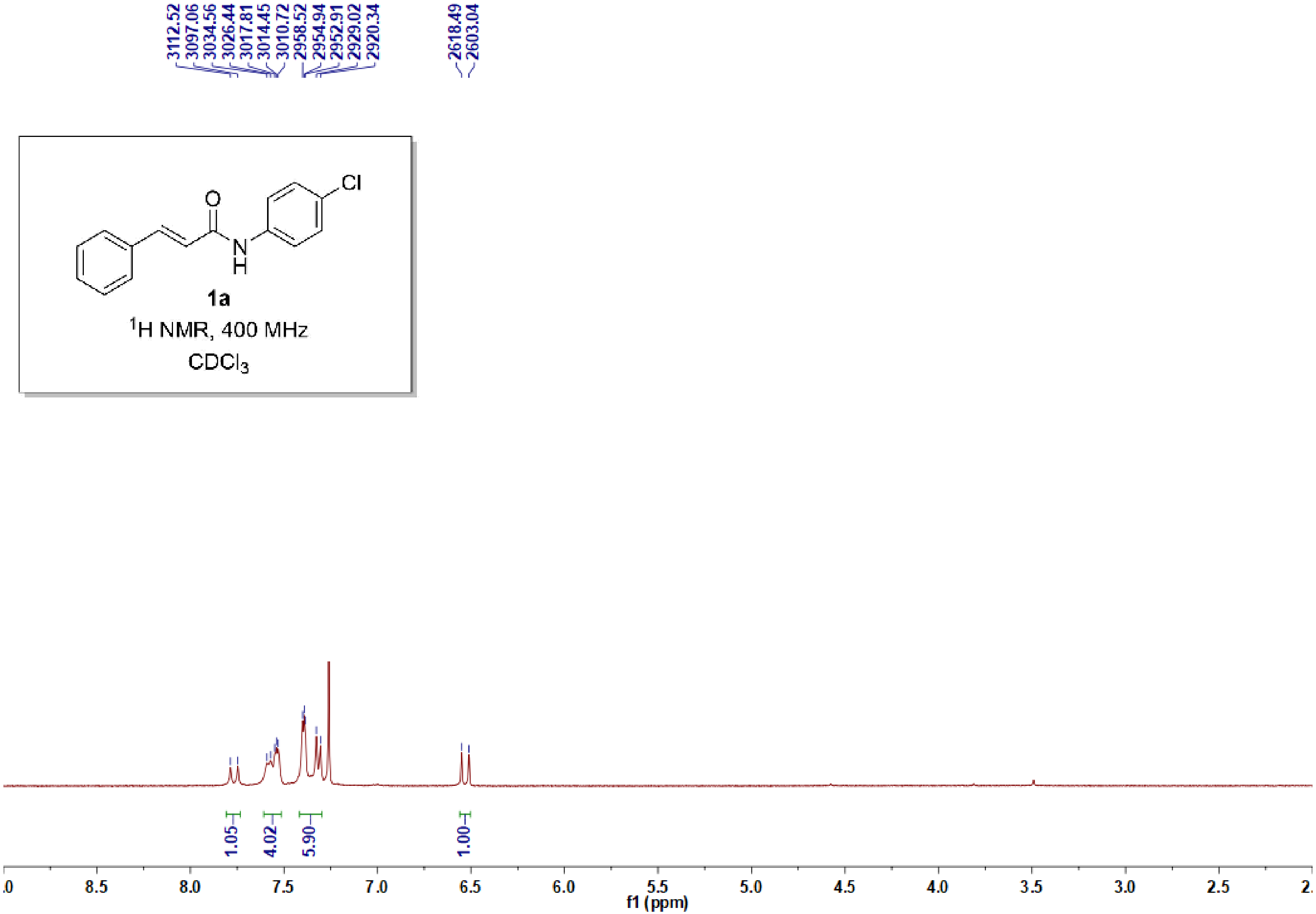
3112.52
3097.06
3034.56
3026.44
3017.81
3014.45
3010.72
2958.52
2954.94
2952.91
2929.02
2920.34
2618.49
2603.04
Cl
O
N
H
1a
1H NMR, 400 MHz
CDCl3
1.05
4.02
5.90
1.00
8.5
8.0
7.5
7.0
6.5
6.0
5.5
5.0
4.5
4.0
3.5
3.0
2.5
f1 (ppm)

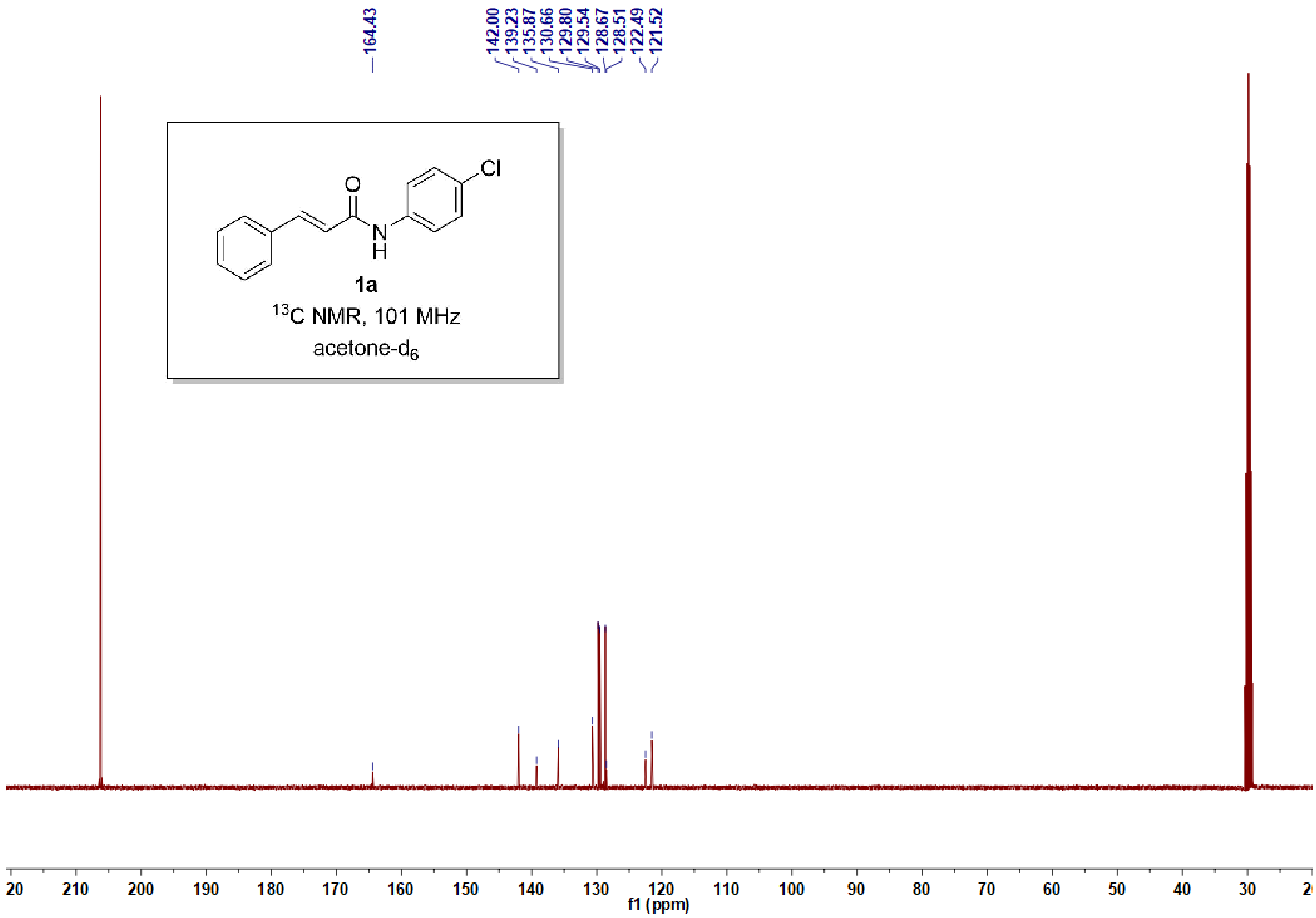
164.43
142.00
139.23
135.87
130.66
129.80
129.54
128.67
128.51
122.49
121.52
Cl
O
N
H
1a
13C NMR, 101 MHz
acetone-d6
f1 (ppm)

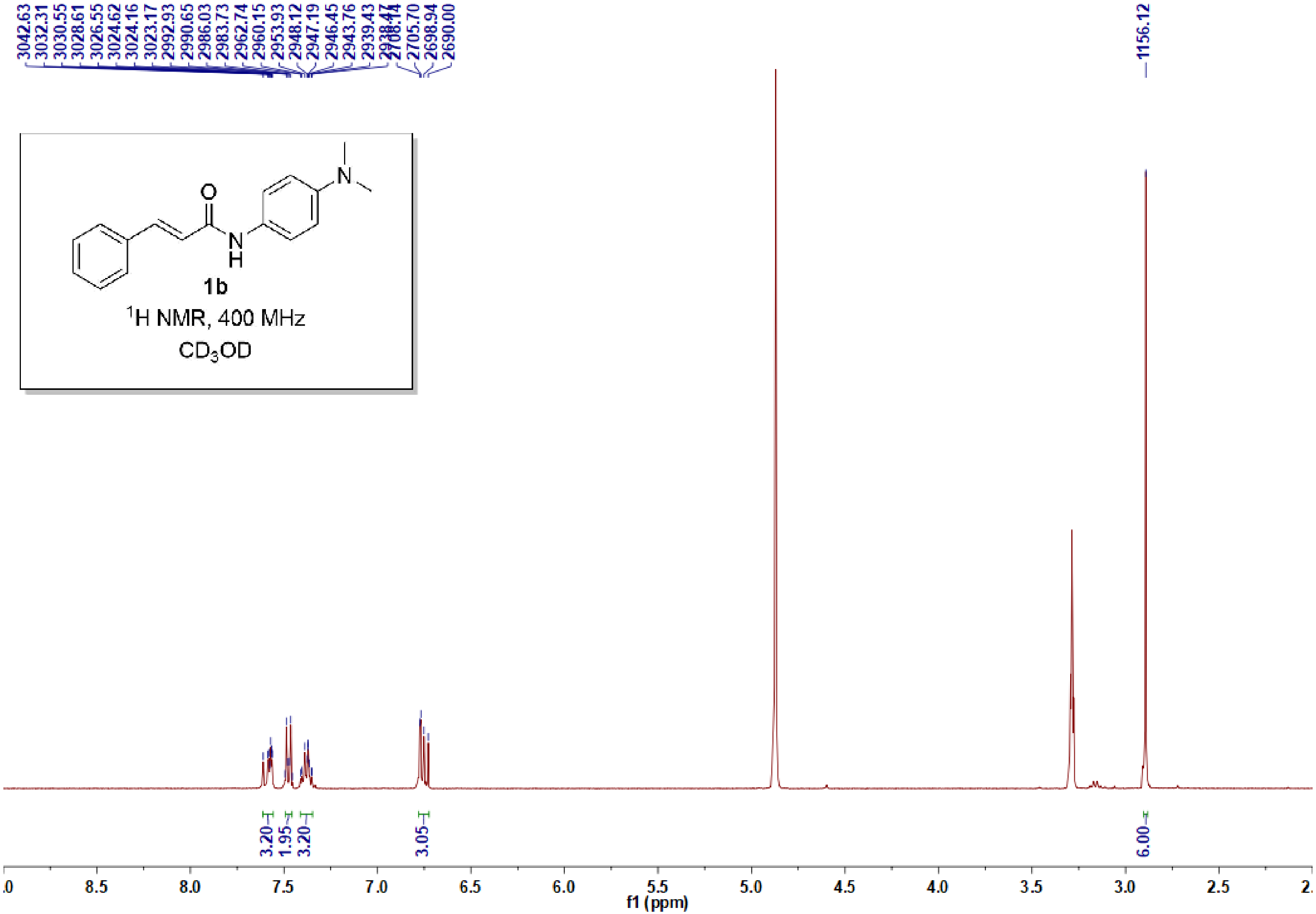
3042.63
3032.31
3030.55
3028.61
3026.55
3024.62
3024.16
3023.17
2992.93
2990.65
2986.03
2983.73
2962.74
2960.15
2953.93
2948.12
2947.19
2946.45
2943.76
2939.43
2705.70
2698.94
2690.00
1156.12
1b
^{1}H NMR, 400 MHz
CD_3OD
3.20
1.95
3.20
3.05
6.00
8.5
8.0
7.5
7.0
6.5
6.0
5.5
5.0
4.5
4.0
3.5
3.0
2.5
f1 (ppm)

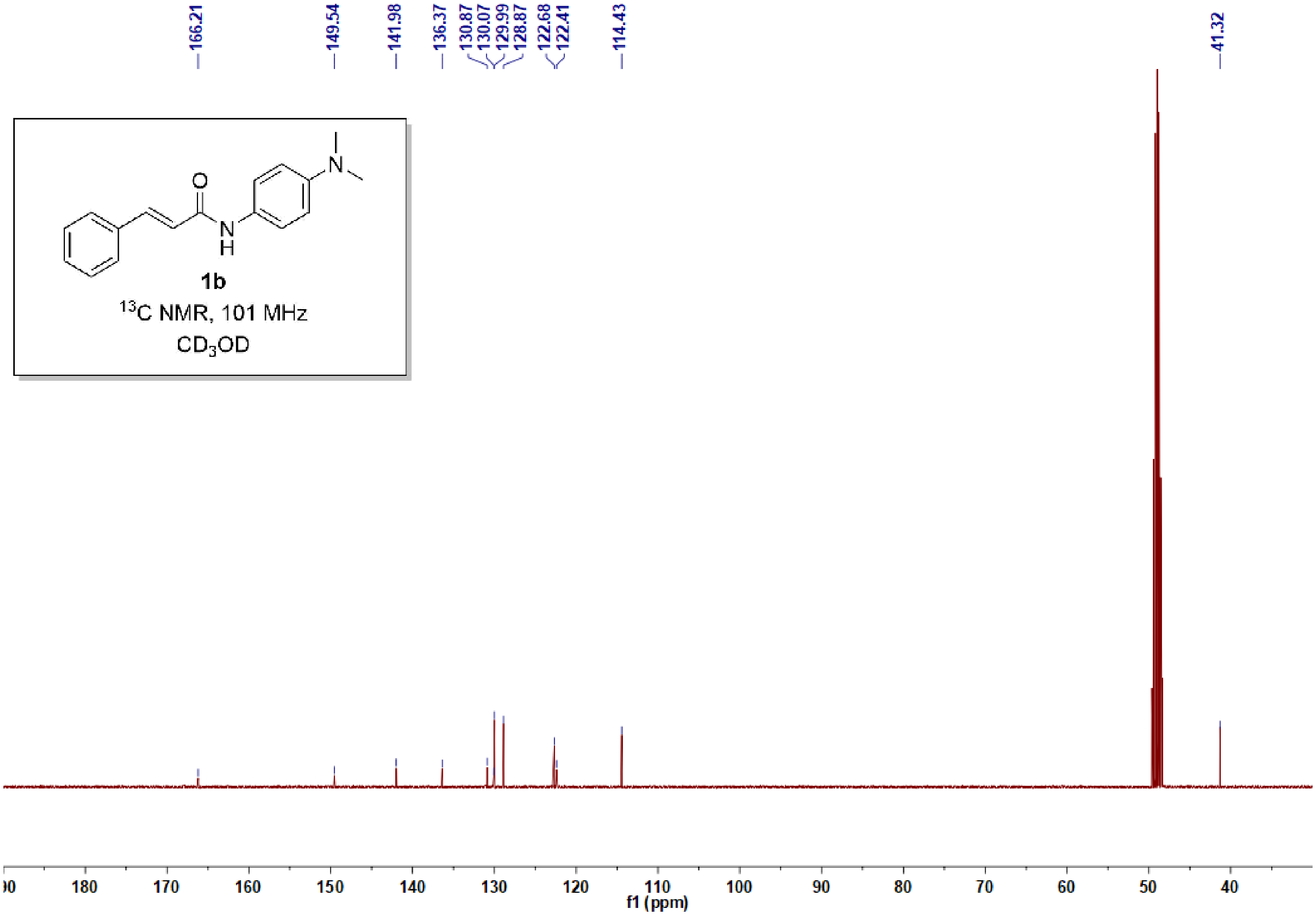
166.21
149.54
141.98
136.37
130.87
130.07
129.99
128.87
122.68
122.41
114.43
41.32
O
N
H
N
1b
^{13}C NMR, 101 MHz
CD_3OD
f1 (ppm)

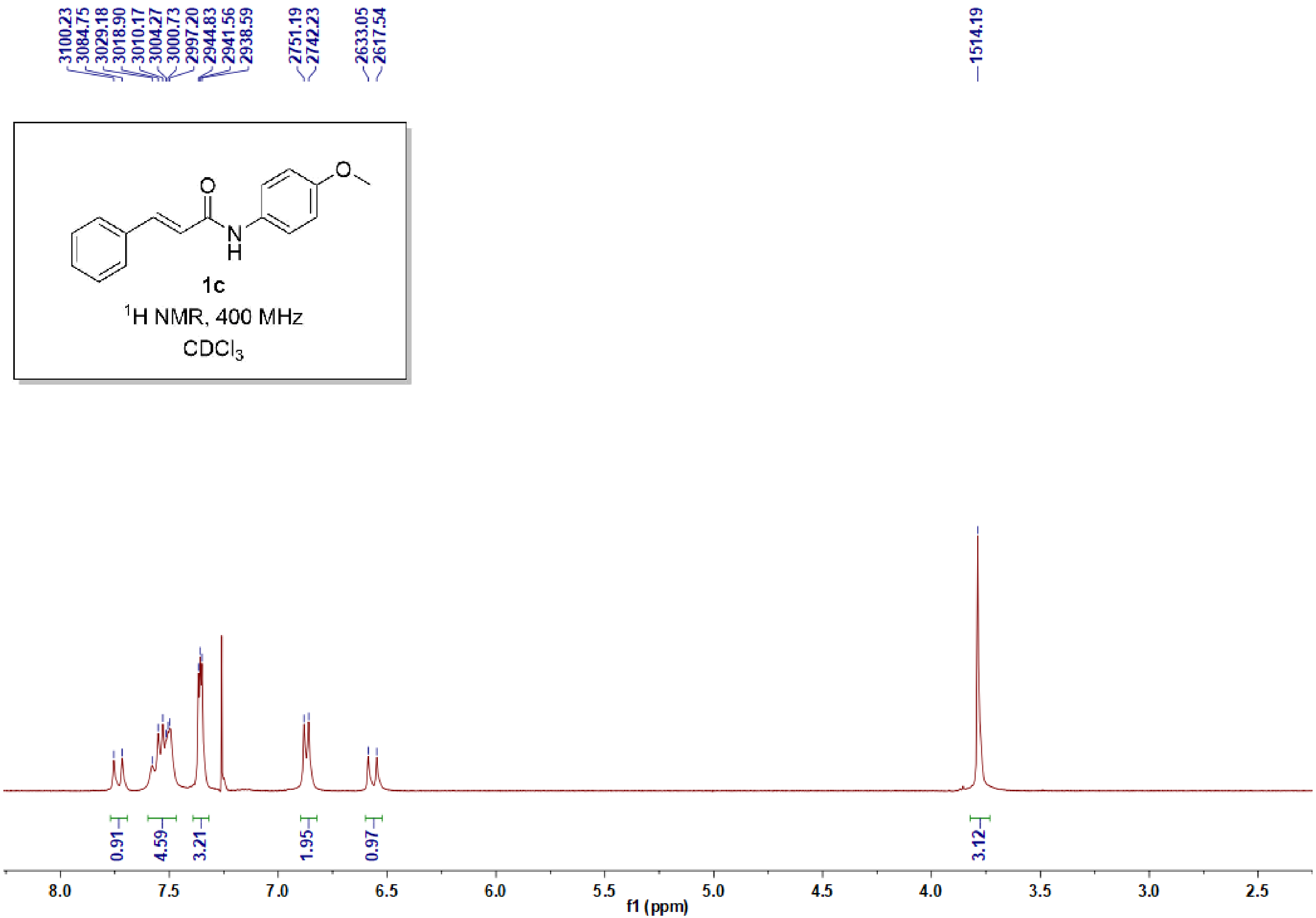
3100.23
3084.75
3029.18
3018.90
3010.17
3004.27
3000.73
2997.20
2944.83
2941.56
2938.59
2751.19
2742.23
2633.05
2617.54
1514.19
1c
^{1}H NMR, 400 MHz
$CDCl_3$
0.91
4.59
3.21
1.95
0.97
3.12
8.0
7.5
7.0
6.5
6.0
5.5
5.0
4.5
4.0
3.5
3.0
2.5
f1 (ppm)

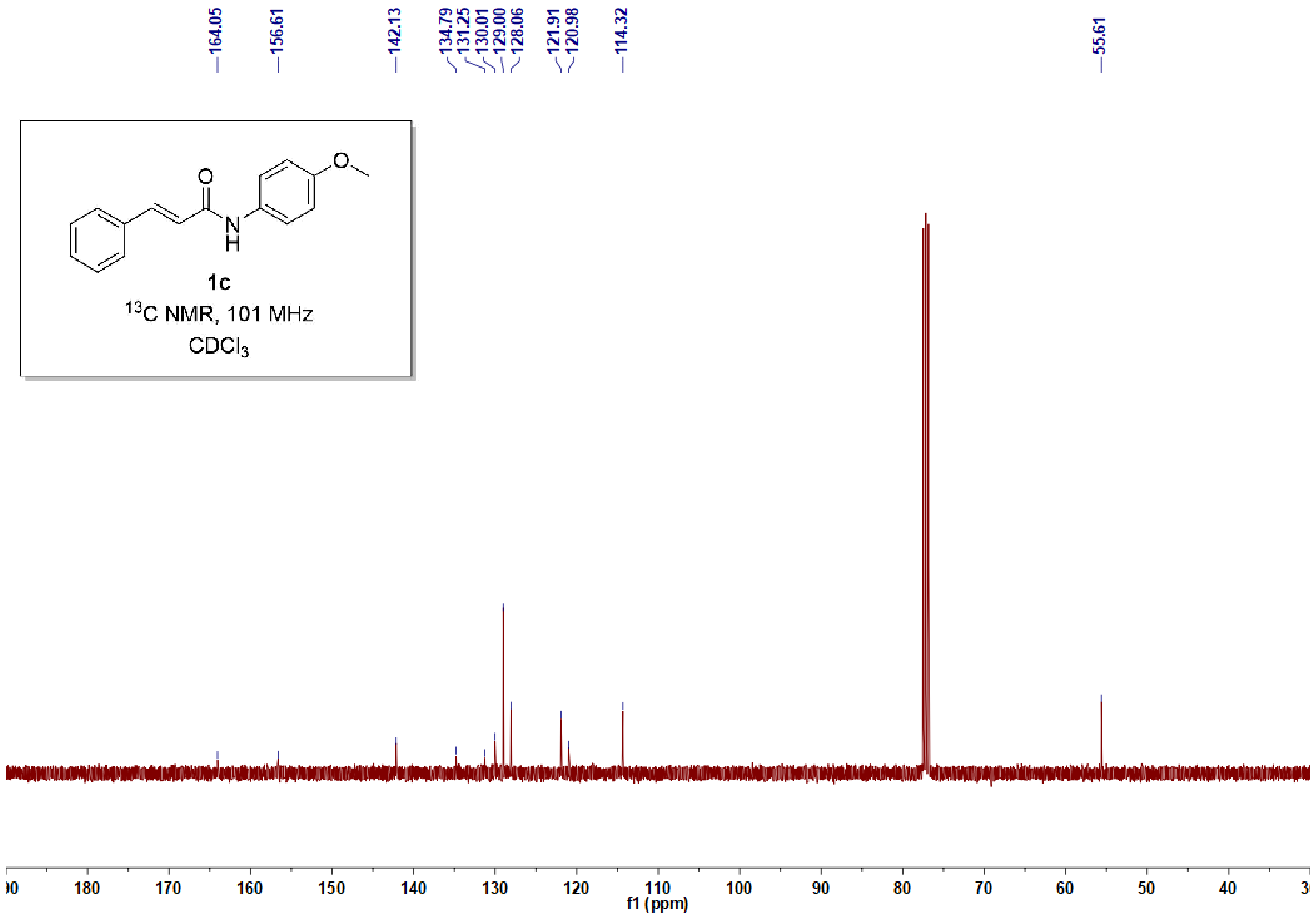
164.05
156.61
142.13
134.79
131.25
130.01
129.00
128.06
121.91
120.98
114.32
55.61
1c
^{13}C NMR, 101 MHz
$CDCl_3$
f1 (ppm)

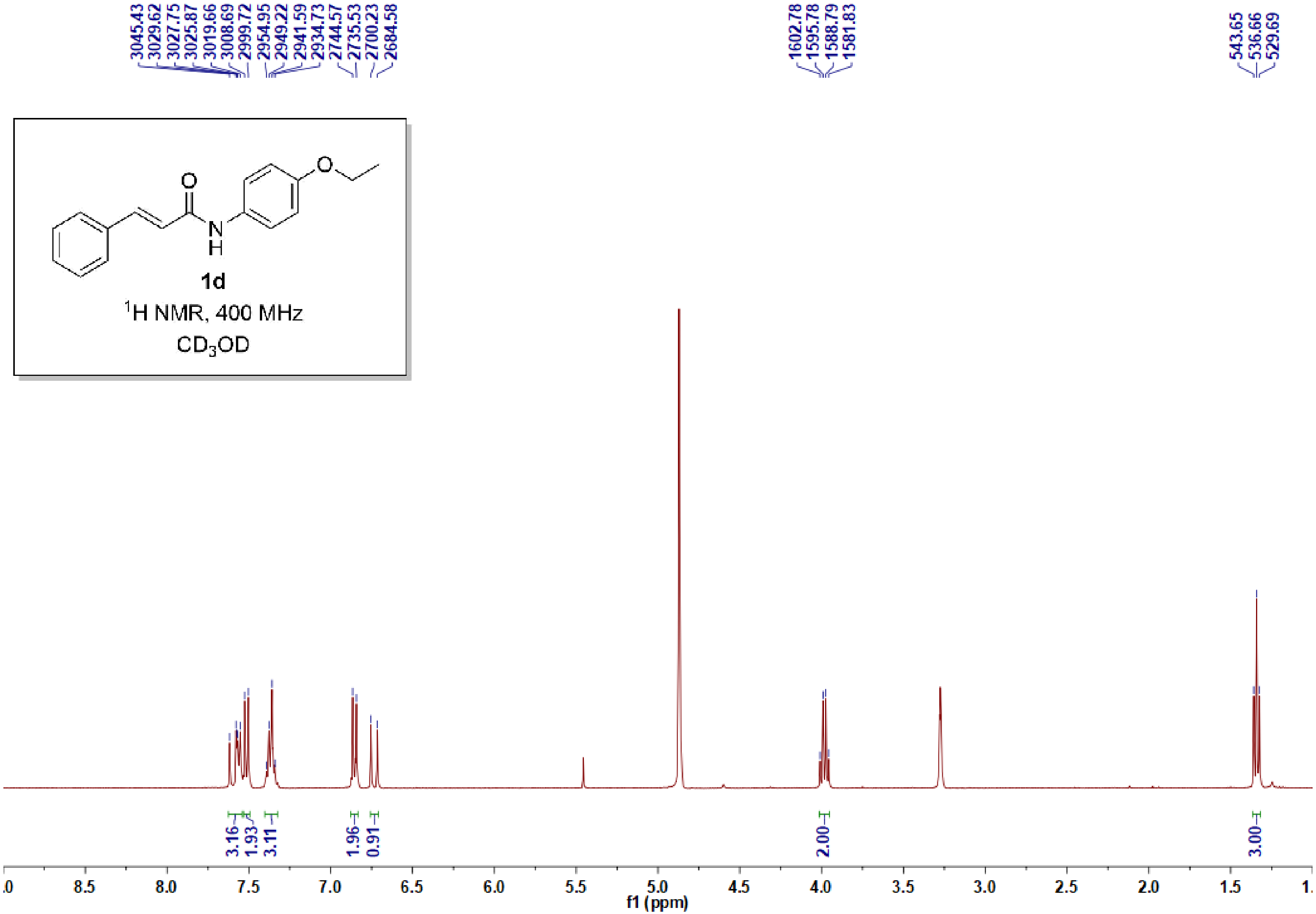
3045.43
3029.62
3027.75
3025.87
3019.66
3008.69
2999.72
2954.95
2949.22
2941.59
2934.73
2744.57
2735.53
2700.23
2684.58
1602.78
1595.78
1588.79
1581.83
543.65
536.66
529.69
1d
^{1}H NMR, 400 MHz
CD_3OD
3.16
1.93
3.11
1.96
0.91
2.00
3.00
8.5
8.0
7.5
7.0
6.5
6.0
5.5
5.0
4.5
4.0
3.5
3.0
2.5
2.0
1.5
f1 (ppm)

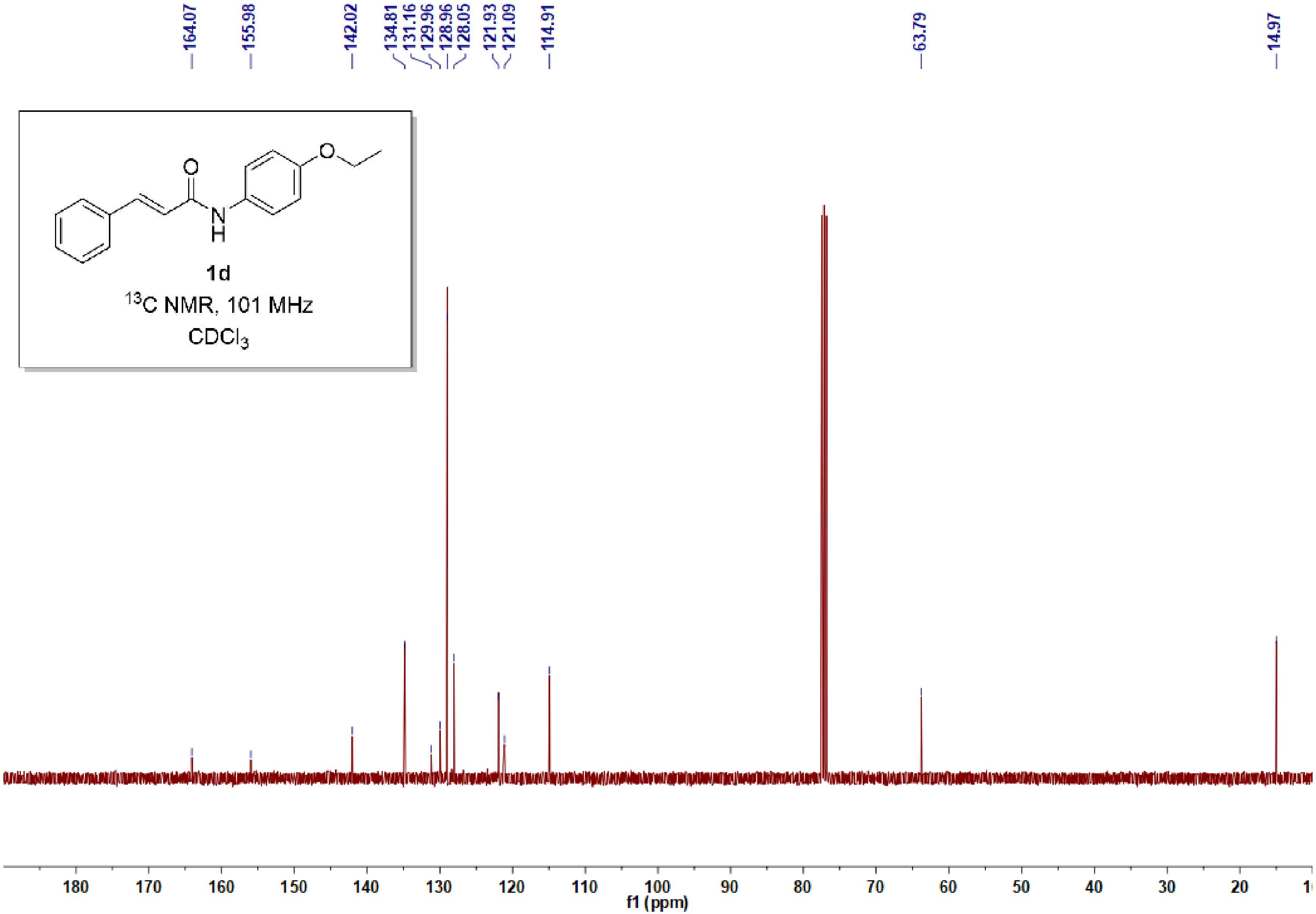
164.07
155.98
142.02
134.81
131.16
129.96
128.96
128.05
121.93
121.09
114.91
63.79
14.97
O
N
H
O
1d
^{13}C NMR, 101 MHz
CDCl$_3$
180
170
160
150
140
130
120
110
100
90
80
70
60
50
40
30
20
f1 (ppm)

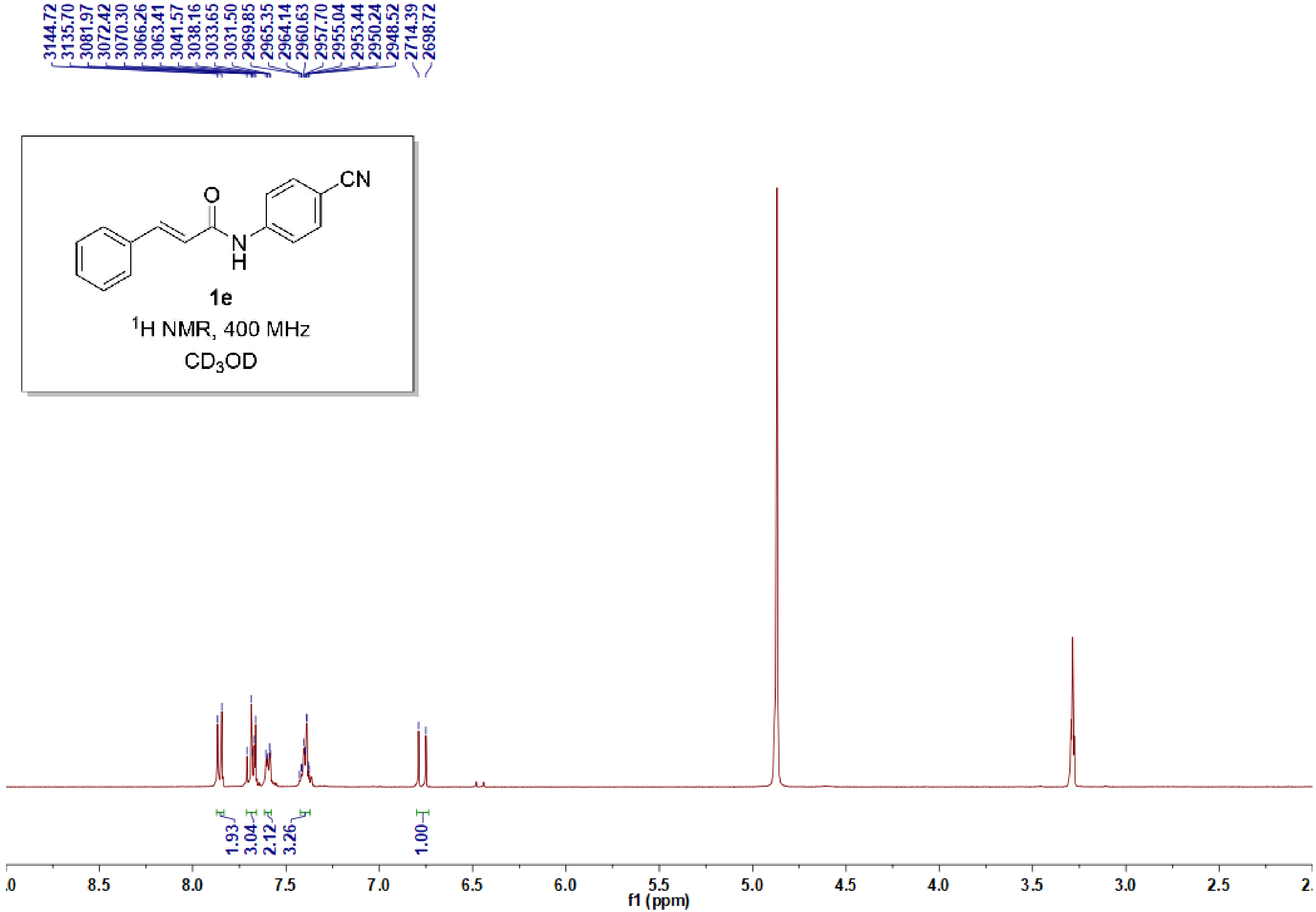
3144.72
3135.70
3081.97
3072.42
3070.30
3066.26
3063.41
3041.57
3038.16
3033.65
3031.50
2969.85
2965.35
2964.14
2960.63
2957.70
2955.04
2953.44
2950.24
2948.52
2714.39
2698.72
CN
O
N
H
1e
^{1}H NMR, 400 MHz
CD_3OD
1.93
3.04
2.12
3.26
1.00
8.5
8.0
7.5
7.0
6.5
6.0
5.5
5.0
4.5
4.0
3.5
3.0
2.5
f1 (ppm)

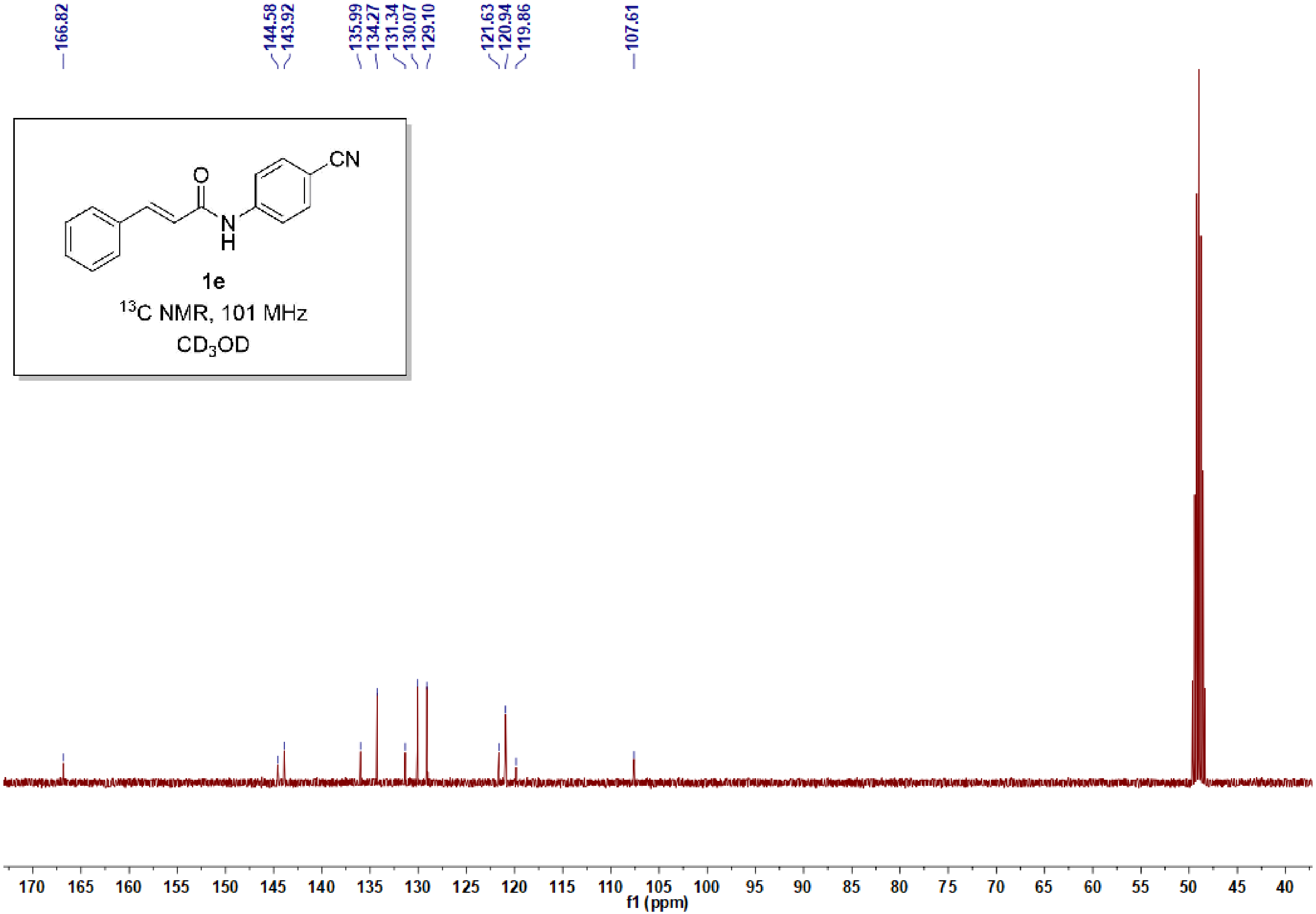
166.82
144.58
143.92
135.99
134.27
131.34
130.07
129.10
121.63
120.94
119.86
107.61
CN
O
N
H
1e
13C NMR, 101 MHz
CD3OD
170 165 160 155 150 145 140 135 130 125 120 115 110 105 100 95 90 85 80 75 70 65 60 55 50 45 40
f1 (ppm)

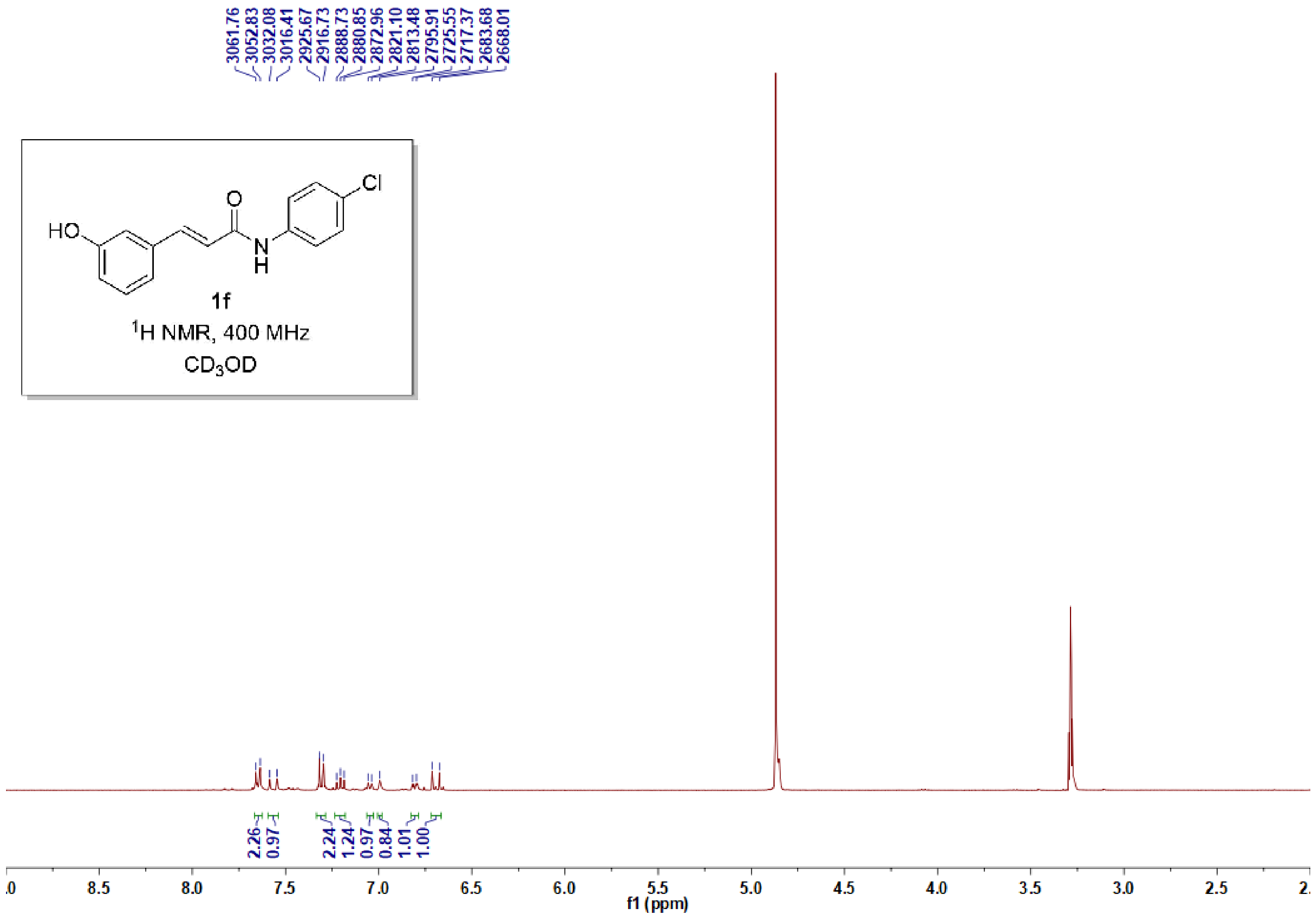
1f
^{1}H NMR, 400 MHz
CD_3OD
f1 (ppm)

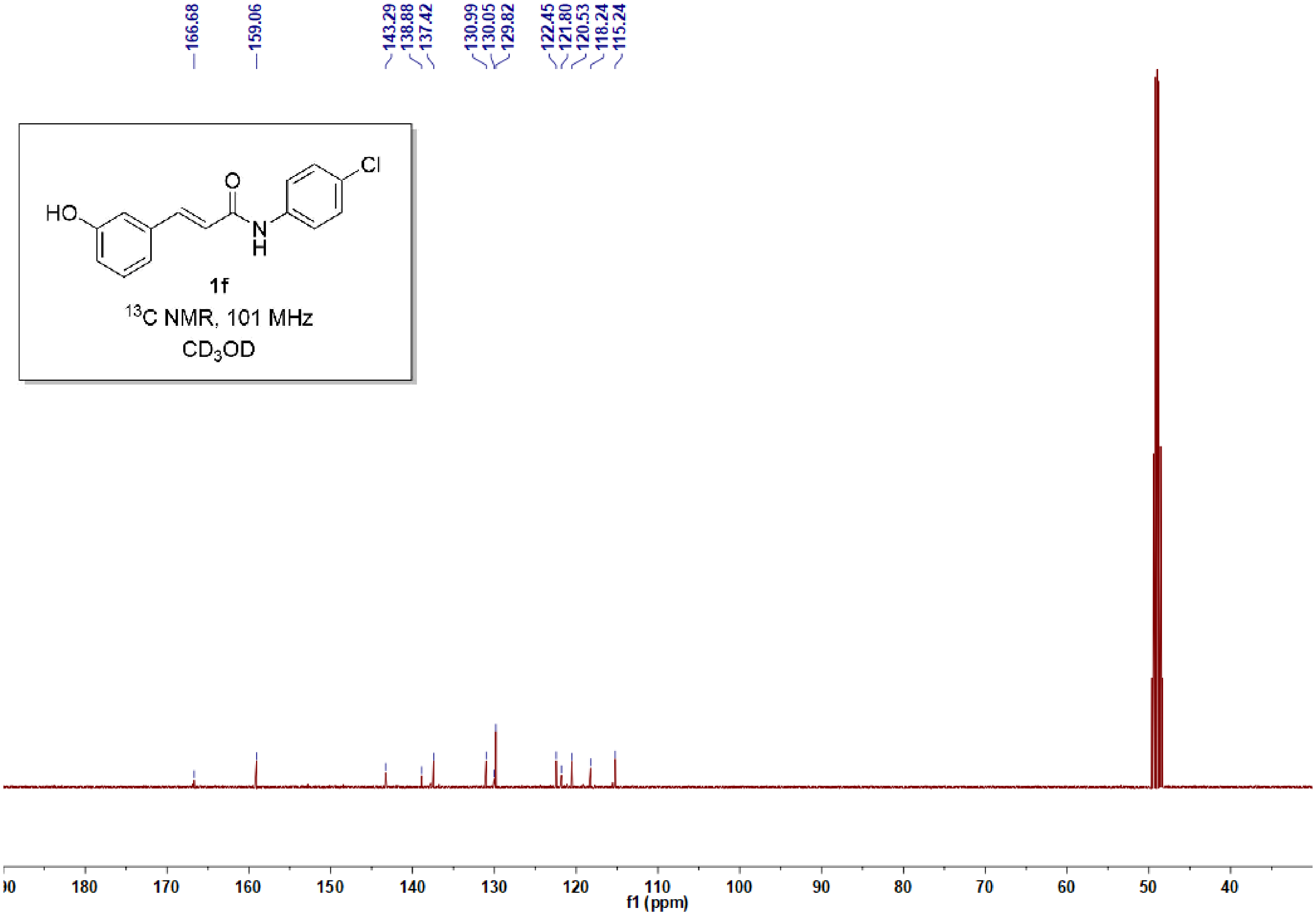
166.68
159.06
143.29
138.88
137.42
130.99
130.05
129.82
122.45
121.80
120.53
118.24
115.24
HO
O
N
H
Cl
1f
13C NMR, 101 MHz
CD3OD
180
170
160
150
140
130
120
110
100
90
80
70
60
50
40
f1 (ppm)

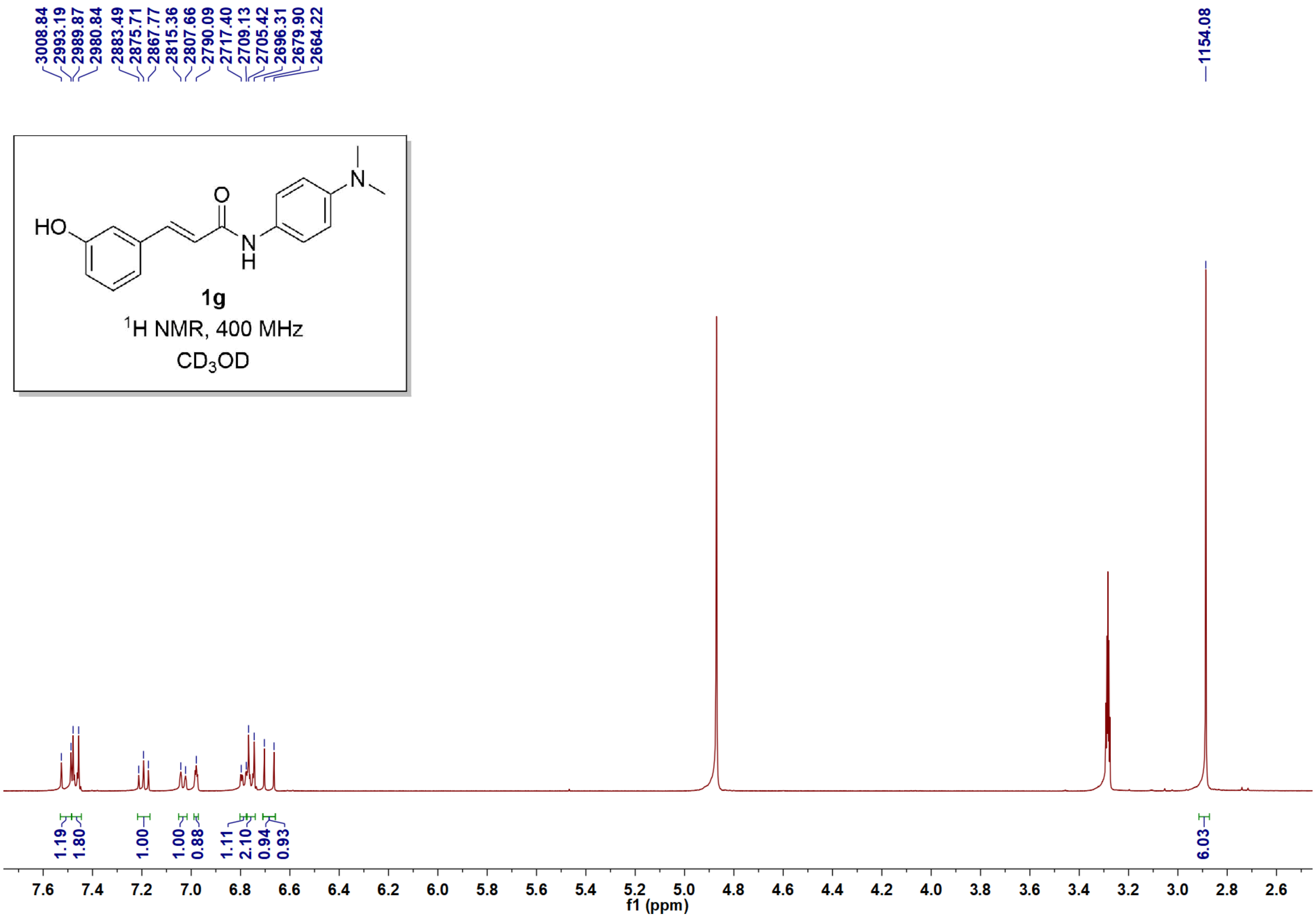
3008.84
2993.19
2989.87
2980.84
2883.49
2875.71
2867.77
2815.36
2807.66
2790.09
2717.40
2709.13
2705.42
2696.31
2679.90
2664.22
1154.08
HO
O
N
H
N
1g
^{1}H NMR, 400 MHz
CD_3OD
1.19
1.80
1.00
1.00
0.88
1.11
2.10
0.94
0.93
6.03
7.6
7.4
7.2
7.0
6.8
6.6
6.4
6.2
6.0
5.8
5.6
5.4
5.2
5.0
4.8
4.6
4.4
4.2
4.0
3.8
3.6
3.4
3.2
3.0
2.8
2.6
f1 (ppm)

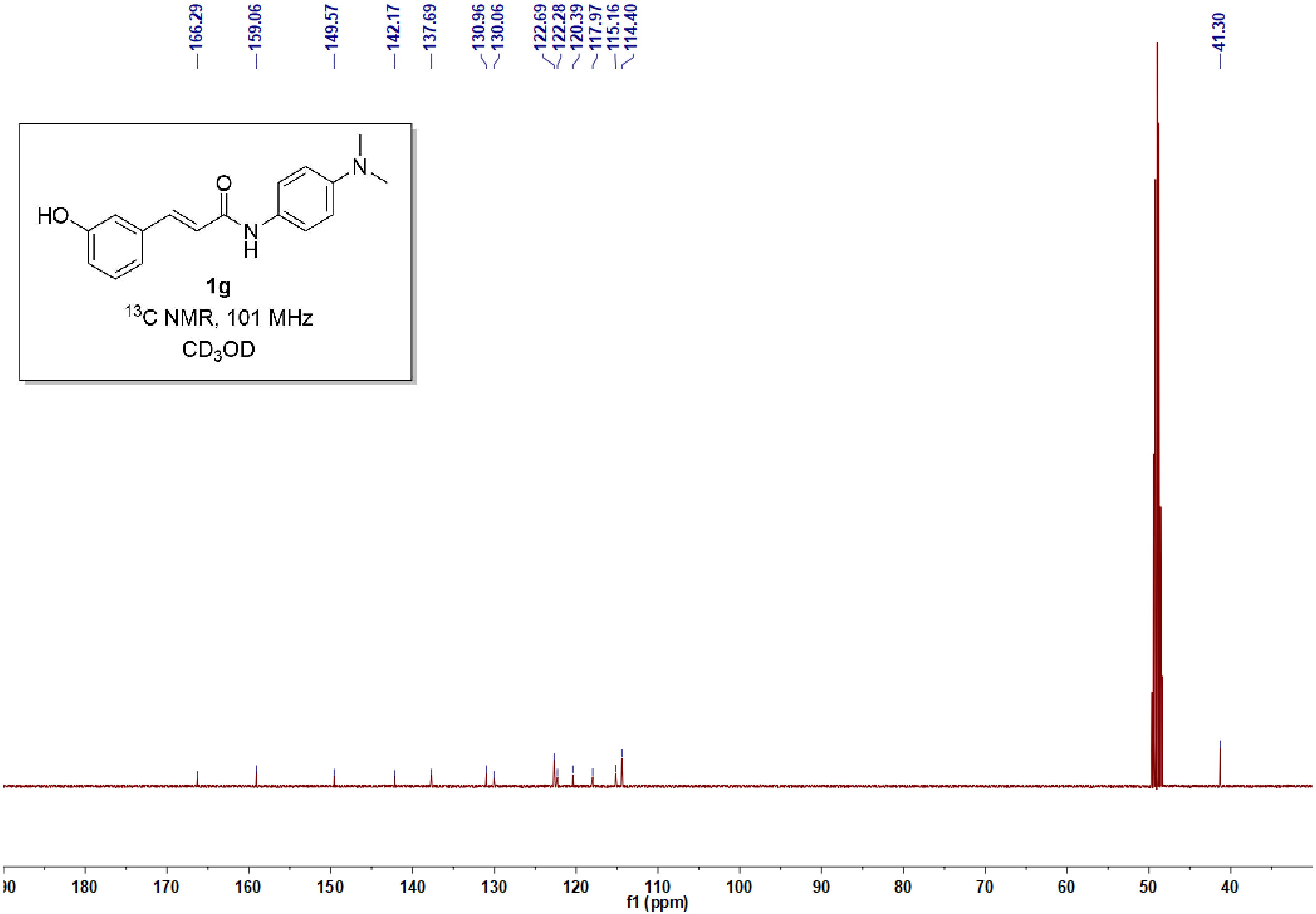
166.29
159.06
149.57
142.17
137.69
130.96
130.06
122.69
122.28
120.39
117.97
115.16
114.40
41.30
HO
O
N
H
N
1g
^{13}C NMR, 101 MHz
CD_3OD
180
170
160
150
140
130
120
110
100
90
80
70
60
50
40
f1 (ppm)

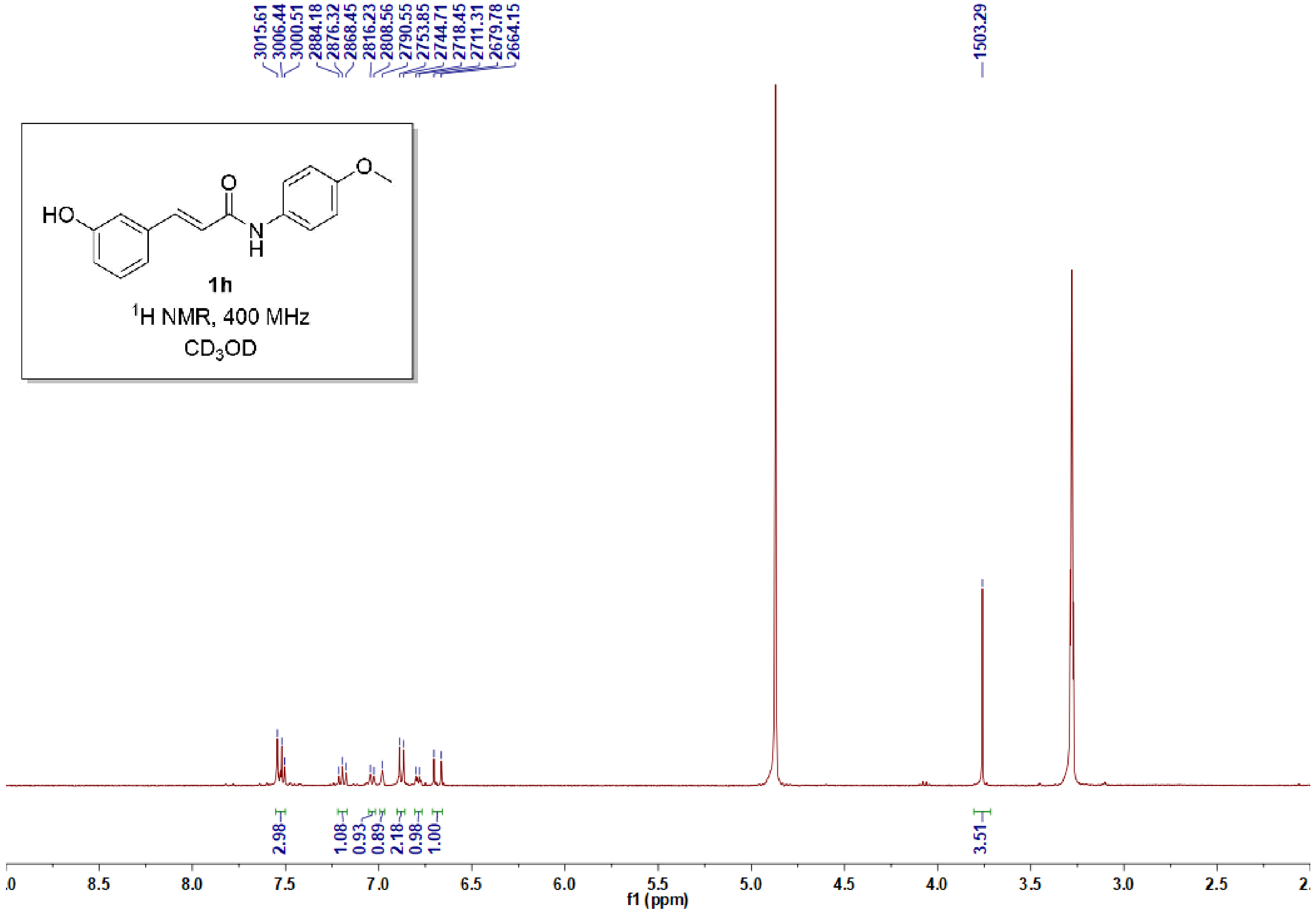
3015.61
3006.44
3000.51
2884.18
2876.32
2868.45
2816.23
2808.56
2790.55
2753.85
2744.71
2718.45
2711.31
2679.78
2664.15
1503.29
HO
O
N
H
O
1h
1H NMR, 400 MHz
CD3OD
2.98
1.08
0.93
0.89
2.18
0.98
1.00
3.51
8.5
8.0
7.5
7.0
6.5
6.0
5.5
5.0
4.5
4.0
3.5
3.0
2.5
f1 (ppm)

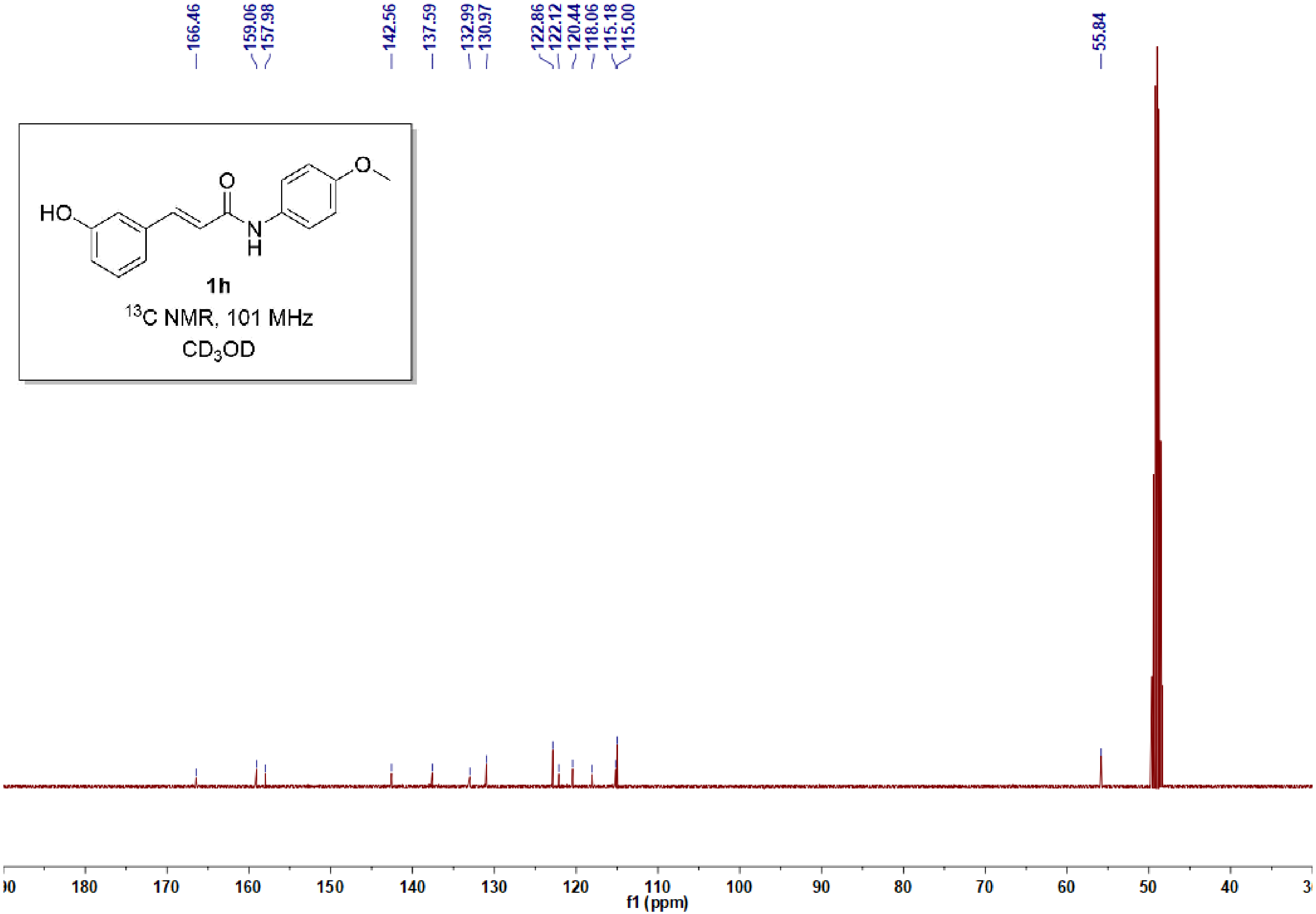
166.46
159.06
157.98
142.56
137.59
132.99
130.97
122.86
122.12
120.44
118.06
115.18
115.00
55.84
HO
O
N
H
O
1h
^{13}C NMR, 101 MHz
CD_3OD
180
170
160
150
140
130
120
110
100
90
80
70
60
50
40
f1 (ppm)

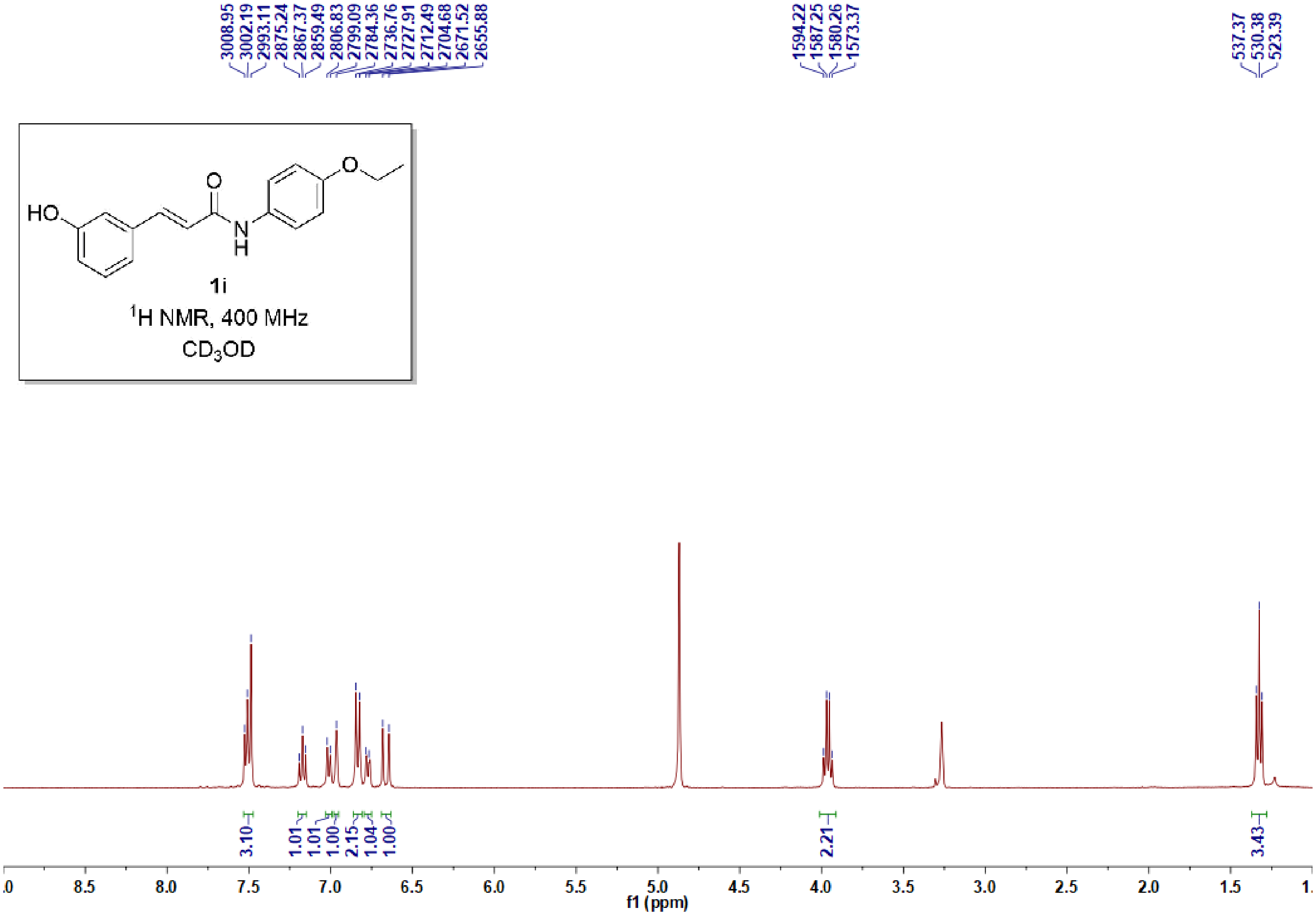
3008.95
3002.19
2993.11
2875.24
2867.37
2859.49
2806.83
2799.09
2784.36
2736.76
2727.91
2712.49
2704.68
2671.52
2655.88
1594.22
1587.25
1580.26
1573.37
537.37
530.38
523.39
HO
O
N
H
1i
1H NMR, 400 MHz
CD3OD
3.10
1.01
1.01
1.00
2.15
1.04
1.00
2.21
3.43
8.5
8.0
7.5
7.0
6.5
6.0
5.5
5.0
4.5
4.0
3.5
3.0
2.5
2.0
1.5
f1 (ppm)

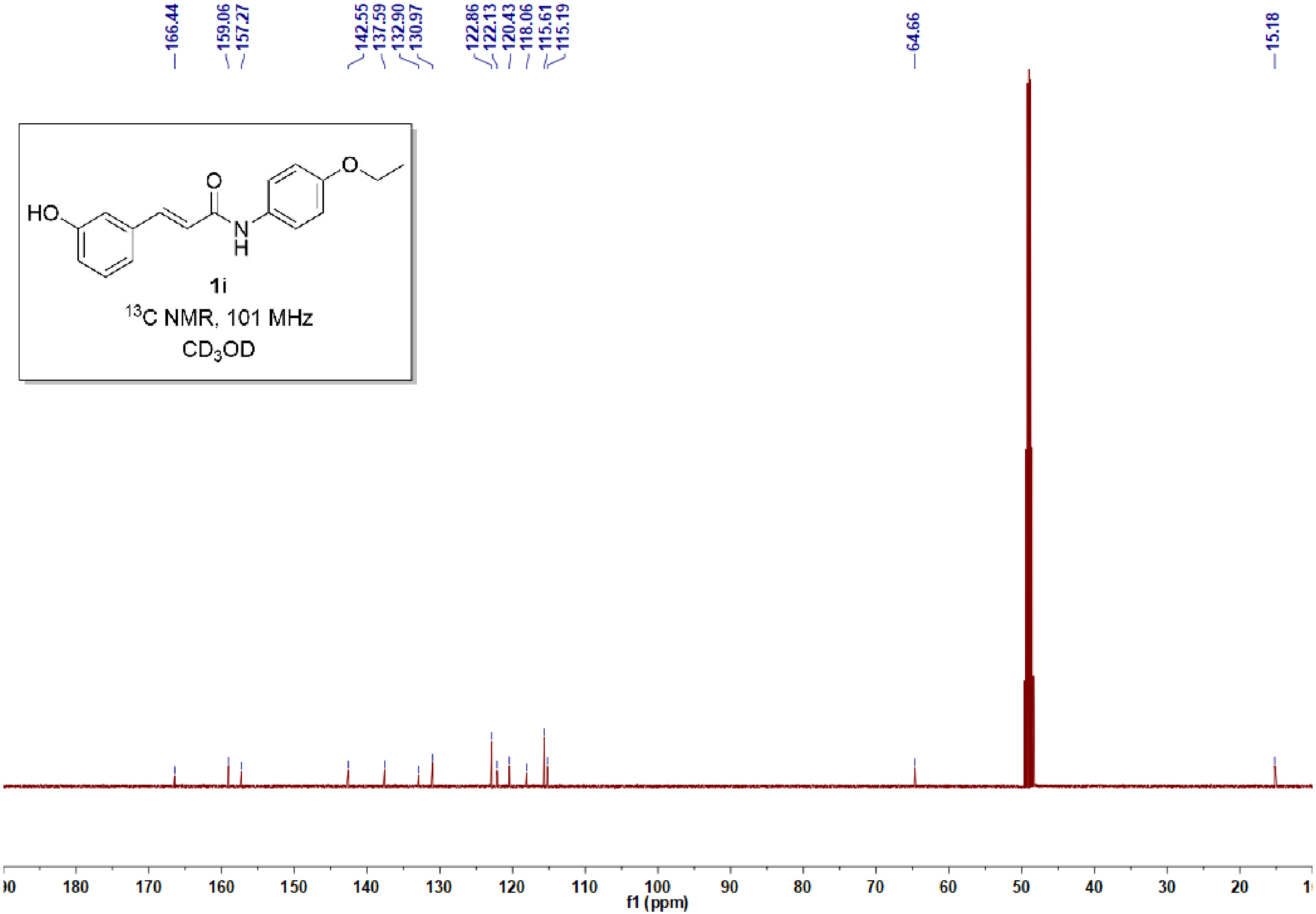
166.44
159.06
157.27
142.55
137.59
132.90
130.97
122.86
122.13
120.43
118.06
115.61
115.19
64.66
15.18
HO
O
N
H
O
1i
^{13}C NMR, 101 MHz
CD_3OD
180
170
160
150
140
130
120
110
100
90
80
70
60
50
40
30
20
f1 (ppm)
